# Development of an Antioxidative Pickering Emulsion Gel through Polyphenol-Inspired Free-Radical Grafting of Microcrystalline Cellulose for 3D Food Printing

**DOI:** 10.1021/acs.biomac.1c00896

**Published:** 2021-10-01

**Authors:** Mahdiyar Shahbazi, Henry Jäger, Rammile Ettelaie

**Affiliations:** † Institute of Food Technology, University of Natural Resources and Life Sciences (BOKU), Muthgasse 18, 1190 Vienna, Austria; ‡ Food Colloids and Bioprocessing Group, School of Food Science and Nutrition, University of Leeds, Leeds, LS2 9JT, United Kingdom

## Abstract

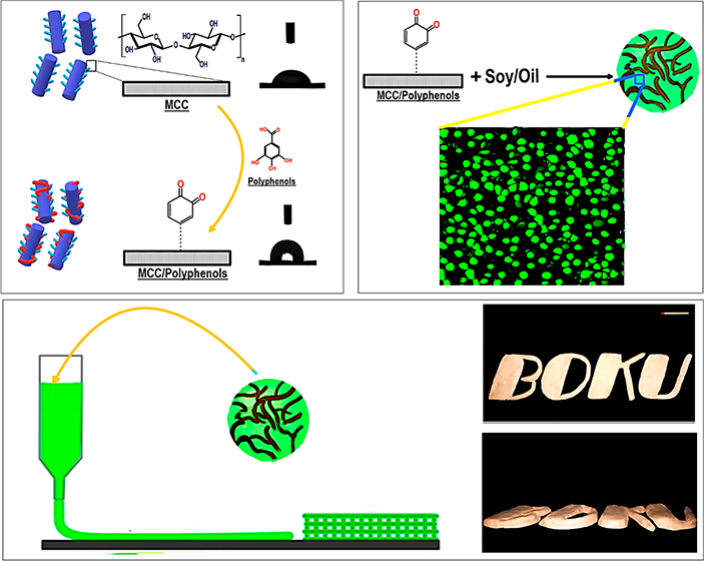

The manufacture of next-generation 3D-printed foods with personalized requirements can be accelerated by in-depth knowledge of the development of a multifunctional biopolymeric-based ink. As a fat replacer in the food industry, microcrystalline cellulose (MCC) has the potential to address the growing need for sustainable healthy reduced-fat 3D printed foods. The modification of MCC structure by polyphenols gives the way to produce a multifunctional antioxidative Pickering emulsion with improved emulsifying properties. In this study, different types of polyphenols, including gallic acid (GA), tannic acid (TA), and cyanidin-3-*O*-glucoside (C3G), were individually used to synthesize the grafted MCC-*g*-polyphenol conjugates by the free-radical grafting method. Then, the antioxidative grafted microconjugates were added to a soy protein-based emulsion gel to partially substitute its oil, and each Pickering emulsion gel variant was printed through an extrusion-based 3D printing system. Emulsifying properties and antioxidant character of MCC were proven to be enhanced after the fabrication of grafted microconjugates. Compared to MCC-*g*-TA, MCC-*g*-GA and MCC-*g*-C3G could efficiently improve the stability of a reduced-fat soy-based emulsion gel upon storage. Moreover, the reduced-fat soy-based emulsion gel containing grafted microconjugates endowed a characteristic shear-thinning behavior with a gel-like structure and superlative thixotropic properties. Following the printing, the antioxidative Pickering emulsion gels containing grafted microconjugates produced well-defined 3D structures with superior lubrication properties. This study demonstrated that the grafting of polyphenols onto MCC could enhance bioactive properties and improve emulsifying performance of MCC, making it a useful component in the development of personalized functional foods.

## 1 Introduction

Three-dimensional (3D) printing has become more important in industry as it offers many advantages in material fabrication, facilitating operative procedures, portable high-precision applications, and customized geometry manufacture of complex architectures. The successful application of 3D printing to create desired printed objects is mostly related to the development of efficient printable ink dispersion.^1^ Commonly, the printable polymeric-based inks must offer well-defined *pseudoplasticity* to provide low flow resistance through nozzles, but also must offer a gel-like structure, possessing sufficient yield stress, to resist compressive stresses from the capillary effects post-printing.^2^

The emulsion stability and printability features of structured gels are the primary quality attributes behind the wide application of emulsion gels in additive manufacturing. Soy proteins are widely served to prepare meat substitutes due to offering a gel-like network. However, because of their poor solubility, dense globular structure, and high molecular weight, soy proteins have low emulsifying ability in comparison with other derived proteins. When they are employed to stabilize an emulsion, only a small portion of soy proteins absorbs onto the droplets, likely as aggregated protein particles.^3^

Because of current advances in emulsion-printing techniques, the adaptability of colloidal particles offers particle-stabilized or “Pickering emulsions” with exclusive functional properties. This type of emulsion is regularly considered to be extremely stable dispersions because of the high energy of particles desorbing from the interface.^4,5^ Thanks to the almost irreversible interfacial adsorption of particulate stabilizers, the highly stable Pickering emulsions also efficiently preserve bioactive compounds inside the oil phase for a long time.^4^ As a sustainable polymer raw material, microcrystalline cellulose (MCC) is often used as a colloidal particle to stabilize O/W emulsion.^4,5^ It is also commonly used as a fat replacer in food products, which can address the growing need for sustainable healthy reduced-fat foods. Though, unmodified MCC with poor surface activities and high hydrophilicity shows a lack of emulsion forming properties, therefore inhibiting its extensive use as a real particulate stabilizer. The results of recent works pointed out the advanced roles of modified MCCs in 3D printing purposes to develop different personalized functional foods.^4,5^ In this regard, tuning the structure of MCC gives a potential technique for realizing the flow behavior of biopolymer-based inks to ones desired for the enhanced printing performance in a given tailored geometry.^2^

Taking advantage of the cost-efficiency, sustainability, and application of eco-friendly reacting agents, there are many endeavors assigned to the polyphenol-inspired grafting modification methods. As an abundant secondary metabolite of plants, the polyphenols, including tannic acid, gallic acid, and cyanidin-3-*O*-glucoside, are considered the main portion of the human diet.^6^ They offer the promise of being useful as a bioactive compound on account of their important and promising physicochemical features. The polyphenols exhibit antioxidant,^6,7^ antibacterial,^8^ anti-inflammatory,^9^ anti-allergic,^10^ and antitumor activities.^9^ Among them, the antioxidant capacity of polyphenols is well documented in the literature.^6^ Besides the bioactivities, numerous research works have stated the applicability of polyphenols as an effective stabilizer of O/W emulsions.^11−13^

Application of high temperatures and shearing force during processing or storage can lead to the thermal and/or oxidative degradation of polyphenols, thus decreasing their functional properties. In this regard, polyphenols with low molecular weight are more susceptible to degradation because of their poor thermal stability. To overcome this limitation, several physicochemical approaches have been introduced to reduce the polyphenols degradation, such as the conjugation of supramolecular biomaterials with polyphenols.^13^ It was stated that the resulting conjugates endow advanced stability with a lower degradation rate compared to components with low molecular weight, preserving the excellent functionalities of polyphenols. Moreover, numerous publications have reported the possible application of therapeutic impacts of polysaccharide–polyphenol conjugates in food industries.^13^ A promising approach to develop the polysaccharide–polyphenol conjugate is the free-radical grafting procedure. This method includes a sustainable and ecofriendly technique that offers grafting of polyphenols onto the biomaterial backbone without the utilization of organic solvent and toxic radical initiators.^14^ To develop the grafting reaction, a green redox pair component, comprising the mixture of ascorbic acid and hydrogen peroxide can be used. The produced radicals can react with polyphenols to promote the development of grafted biopolymer–polyphenol conjugates. These conjugates can be used in the pharmaceutical and food sectors in keeping the functional properties of polyphenols upon processing and storage.

To the best of our knowledge, no reports have shown the utilization of modified multifunctional MCC to develop a stable antioxidative Pickering emulsion gel for 3D food printing. In this paper, first, the functionalized MCC conjugates were produced by grafting different types of polyphenols using the free-radical grafting method with hydrogen peroxide/ascorbic acid redox pair. The utilization of this redox system endows the grafting of MCC to be accomplished with high reaction yields and without the development of toxic compounds. Next, each grafted MCC–polyphenol conjugate, as a fat replacer, was individually added to a soy protein-based emulsion gel to partially substitute its oil, developing an antioxidant-stable Pickering emulsion gel. Finally, we focus on the last category of low-fat 3D printed meat analogue products, where reduced-fat Pickering emulsion gel stabilized by grafted microconjugates was printed through a 3D printer in a layer-by-layer fashion.

## 2 Methods and Materials

### 2.1 Materials

SPI isolate (SPI) (moisture: 4.83%, fat: 0.32%, protein: 92.88%, ash: 3.40%, pH: 7.09, and viscosity of 1% wt. solution: 10 cP) was obtained from Archer Daniels Midland Company (ADM, Decatur, IL). Microcrystalline cellulose (MCC) Avicel PH-101 was purchased by Sigma (Sigma-Aldrich GmbH, Sternheim, Germany). Gallic acid (GA), tannic acid (TA), cyanidin-3-*O*-glucoside (C3G), and Tripyridyl-*S*-triazine (TPTZ) were supplied from Sigma-Aldrich (St. Louis, MO). Folin-Ciocalteau’s phenol reagent and 2,2-diphenyl-1-picrylhydrazyl (DPPH) were purchased from MP Biomedicals (Irvine, CA). l-Ascorbic acid and hydrogen peroxide (H_2_O_2_) were obtained from Sigma (Sigma-Aldrich GmbH, Sternheim, Germany). All other reagents used were analytical grade without further purification.

### 2.2 Synthesis of Grafted MCC–Polyphenol Conjugates (Grafted Microconjugates)

MCC was completely dispersed in distilled water (100 g L^–1^) and stirred using a magnetic stirrer for 120 min at ambient temperature. Then, the MCC suspension was sheared using a high-speed rotor-stator device (Ultra-Turrax, IKA* T25 digital, Germany) for 20 min, which generated shear force at a shear rate of 210 s^–1^ (5690 G-force). After completing the process, the sheared MCC was collected and dried in an oven at 40 °C for 36 h. After that, the dried MCC was ground to disrupt the clumps, and filtered by a sieve to attain the particle size of 20 μm.

The synthesis of grafted microconjugates, including MCC-*g*-GA, MCC-*g*-TA, and MCC-*g*-C3G, was performed through the free-radical grafting method. In this regard, the pretreated MCC (5 g) was dispersed in 100 mL of distilled water (5 wt %) and stirred through a high-speed rotor-stator device (Ultra-Turrax, IKA* T25 digital, Germany) at 50 °C for 120 min to obtain a homogeneous dispersion. Next, 5 mL H_2_O_2_ (1.0 M) containing 0.08 g of ascorbic acid was introduced to the MCC-based dispersion. Then, the MCC dispersion containing redox initiator compounds was homogenized via an ultrasonic cleaning device (Bandelin 400, Berlin, Germany), operating at 15 kHz for 2 min. Finally, the different mass ratio from each polyphenol compound to MCC (0.5:1, 1:1, 1.5:1, and 2:1) was individually incorporated into the reaction vessel and vigorously stirred at 28 °C for 48 h to ensure complete hydration. Then, the product was centrifuged (Eppendorf centrifuge 5417R, Hamburg, Germany) at 1409 G-force for 20 min in the ambient condition and washed five times with deionized water. Finally, the supernatant was dried using a freeze-dryer device (Christ Alpha 1–2LD plus, Germany) to obtain a well-separated particle. Blank MCC, as a control, was obtained in similar circumstances albeit without the polyphenols.

### 2.3 Characterization of Grafted Microconjugates

#### 2.3.1 Fourier-Transform Infrared Spectroscopy (FTIR)

The transmission infrared spectra of pristine MMC and grafted microconjugates were identified with an FTIR spectrometer (Jasco FT/IR6200, Tokyo, Japan) to confirm the grafting process. The solid samples needed for the FTIR assay were obtained in the pellet form by blending approximately 10 mg of each sample with 100 mg of dry potassium bromide (KBr). Next, the samples were transferred to pellets to scan the spectral area at the wavenumber ranges of 400 and 4000 cm^–1^, in which 50 scans were recorded with 1 cm^–1^ resolution.^4^

#### 2.3.2 Solid-State ^13^C (NMR) Spectroscopy

To further verify the grafting of MCC structure by polyphenol variants, solid-state ^13^C NMR was performed through a Bruker spectrometer (AvanceIII 500, Bruker, Ettlingen, Germany) equipped with a 4 mm MAS (magic angle spinning) probe, where frequency for carbons and protons was 75.46 and 300.13 MHz, respectively. The glycine as an external reference was utilized to obtain the ^13^C spectra and to set the Hartmann–Hahn matching condition in the cross-polarization experiments. The spectrum of each sample was obtained with the ramp {^1^H} → {^13^C} cross-polarization (CP)/MAS pulse sequence using the proton decoupling upon acquisition. The recycling period was 10 s and a contact time of 3 ms during CP was adjusted for all experiments.^4^

#### 2.3.3 Determination of the Degree of Grafting

The content of each polyphenol in the synthesized grafted microconjugates was measured based on the verified methods of Folin–Ciocalteu^15^ with slight modification. In short, the freeze-dried MCC–polyphenol conjugate variants (5 mg mL^–1^) were dispersed in distilled water and stirred at 30 °C for 60 min. Next, 0.5 mL of suspension was blended with 1 mL of Folin–Ciocalteu reagent (5 times dilution) and was continuously stirred for 60 min in the dark. The reaction was initiated by introducing 5 mL sodium carbonate (10 w/v%). The suspension was treated by a high-speed rotor-stator device (Ultra-Turrax, IKA* T25 digital, Germany) for 2 min, which induced a shear force at a shear rate of 400 s^–1^ (20664 G-force). The reaction was accomplished within 18 h in ambient temperature and under atmospheric pressure. Finally, the absorbance of the homogeneous dispersion was measured at 650 nm through a UV–vis spectrophotometer (UV-2550, Shimadzu, Japan). GA, TA, and C3G were used as a standard and the grafting degrees of the grafted microconjugate variants were expressed as milligrams of each polyphenol equivalent per gram of grafted MCC–polyphenol conjugate.

#### 2.3.4 Transmission Electron Microscopy (TEM)

To evaluate the morphology of the grafted microconjugate variants, the TEM was performed on a Hitachi-7650 TEM (Hitachi Co., Ltd., Japan) at an acceleration voltage of 80 kV. The particle aqueous suspension (0.05 wt %) was placed onto a Formvar-coated 200-mesh TEM grid (Canemco Inc., Canada) and dried at room temperature before observation.

#### 2.3.5 X-ray Diffraction (XRD)

The XRD diffractogram was obtained through an X-ray diffractometer (Shimadzu XRD 7000, Tokyo, Japan) with Cu Kα irradiation. The samples were exposed to the X-ray beam at 2θ angles ranging from 2° to 60° running at 45 kV and 40 mA, employing Cu Kα radiation (λ = 1.541 Å) at a speed of 2° min^–1^. To evaluate the relative crystallinity degree (RCD), total curve area (*I*_t_), and the area under the peaks (*I*_p_) were determined using the software offered by Shimadzu, and RCD was measured from eq 1:^16^
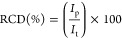
1

#### 2.3.6 Water Contact Angle

The contact angle assay was performed through an OCA 20 contact angle meter (Dataphysics Instruments GmbH, Filderstadt, Germany) using the sessile drop approach. A uniform thin film was fabricated using KW-4A spin-coater (CHEMAT Technology Northridge, CA) through spin coating 2.0 wt % pristine and modified MCCs (in toluene) suspensions onto the silicon wafers at a shear rate of 210 s^–1^ (5690 G-force) for 1 min, followed by heat treatment at 90 °C for overnight. Then, the obtained films were sectioned into the rectangular strips of (4 × 6) cm^2^ and placed on a horizontal movable stage. A drop (5 μL) of deionized water with a Hamilton syringe (10 μL, Hamilton, Switzerland) was deposited centrally on the surface of the films. The value of CA was measured in natural light after 30 s.^17^ The data were analyzed by the relevant software offered by the Dataphysics Instruments.

#### 2.3.7 Scavenging Activity on DPPH Free-Radicals

The DPPH free-radical test has been extensively applied to evaluate the preliminary radical scavenging capacity of plant extracts or antioxidant compounds.^18^ In this regard, the antioxidant evaluation was conducted by DPPH radical scavenging experiment. The DPPH solution was obtained by introducing 5.0 mg of DPPH in methanol (100 mL). The aqueous suspensions/solutions of pristine MCC and grafted microconjugate variants were individually prepared by dispersing 50.0 mg of each sample in 100 mL of deionized water and stirred for 60 min. After introducing 5 mL of the DPPH solution to each tube, the resulting mixtures were shaken vigorously and were incubated at ambient temperature in the dark for 60 min. Next, the reactants were centrifuged (Eppendorf centrifuge 5417R, Hamburg, Germany) at 4000 G-force for 5 min. Afterward, the absorbance was measured at 517 nm using a UV–vis spectrophotometer (UV-2550, Shimadzu, Japan). The ascorbic acid was utilized as the positive control. The scavenging effect of DPPH radical was measured as follows:
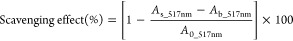
2where *A*_0_517 nm_ is the absorbance of the control (using deionized water instead of the sample), *A*_s_517 nm_ is the absorbance of the samples mixed with reaction solution, and *A*_b_517 nm_ is the absorbance of the sample under the same condition as *A*_s_517 nm_, but ethanol was used instead of ethanol solution of DPPH.

#### 2.3.8 Total Antioxidant Capacity

The ferric-reducing antioxidant potential was used to determine the total antioxidant capacity. The ferric reducing antioxidant potential working solution was provided by mixing 100.0 mL of sodium acetic buffer (0.3 M, pH 3.6), 10.0 mL of TPTZ (10 mM, dissolved in 40 mM HCl), and 10.0 mL of FeCl_3_ solution (20 mM) together. The mixture containing 50 μL of sample and 100 μL of the ferric reducing antioxidant potential solution was incubated at room temperature for 20 min, and the absorbance was detected at 593 nm.
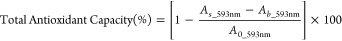
3where *A*_0_593 nm_ is the absorbance of the control (using deionized water instead of the sample), *A*_s_593 nm_ is the absorbance of the samples mixed with a working solution, and *A*_b_593 nm_ is the absorbance of the sample under the same condition as *A*_s_593 nm_, but ethanol was used instead of working solution.

### 2.4 Preparation of SPI-Based Pickering Emulsion

The SPI aqueous dispersion was obtained by dispersing SPI powder (25.0 g) into part of the citrate phosphate buffer (pH 5.6, 60 mL), with the rest of the water being used for the grafted microconjugates. Then, the SPI-based dispersion was stirred at 45 °C for 80 min through a magnetic heater stirrer. Simultaneously, canola oil (10% (v/v)) was added to the dispersion. The obtained emulsions were stirred by an Ultra-Turrax with a shear rate of 400 s^–1^ (20664 G-force) for 5 min. Separately, a stock suspension from pristine MCC or each grafted microconjugate (i.e., MCC-*g*-GA, MCC-*g*-TA, and MCC-*g*-C3G) was prepared by dispersing the weighed amount (70 wt %) of the powdered pristine MCC or grafted microconjugates into the phosphate buffer (pH 5.6, 40 mL). The prepared dispersions were mixed with a high-speed rotor-stator device (Ultra-Turrax T25D IKA, Germany) at a shear rate of 400 s^–1^ (20664 G-force) for 10 min at ambient temperature. Next, the pristine MCC or grafted microconjugates suspensions were gently stirred overnight at room temperature. The pH of the obtained suspensions was then adjusted back to pH 5.6.

An O/W emulsion was prepared by blending canola oil 10 and 90 wt % aqueous SPI-based dispersions using a high-speed blender (Ultra-Turrax T25D IKA, Germany) for 5 min. This coarse emulsion was homogenized by a two-stage high-pressure Microfluidizer processor (M110-PS, Microfluidics international Corp., Newton, MA) with 1800 psi at the first stage and 700 psi at the second stage. The full-fat stabilized emulsion regarded as control hereafter (10 wt % canola oil, 25.0 wt % SPI, pH 5.6) was employed to develop reduced-fat inks. After that, a 60% reduced-fat SPI-based Pickering emulsion gel was prepared by replacing oil with the stock suspensions of pristine MCC (SP/MC) or MCC-*g*-GA (SP/MC/GA), MCC-*g*-TA (SP/MC/TA), and MCC-*g*-C3G (SP/MC/C3G) (70 wt %, pH 5.6) (see Supporting Information, Section S.2). The reduced-fat emulsions contained 4 wt % canola oil and 4.2 wt % of pristine MCC or grafted microconjugate variants. In all formulations, the level of SPI was considered constant (25.0 wt %). All Pickering emulsions were conditioned in a controlled biochamber (ACS Sunrise 700 V, Alava Ingenieros, Madrid, Spain) at 25 °C with a relative humidity of (37 ± 1)% for 48 h.

### 2.5 Characterization of SPI-Based Inks

#### 2.5.1 Emulsion Stability

The stability experiment was performed by a Turbiscan Lab Expert stability analyzer (Formulaction, Toulouse, France) for 180 min at ambient conditions. The emulsions stability was carried out according to multiple light backscattering of a pulsed near-infrared light (880 nm). First, the inks were moved to a tested bottle attaining a height of 42 mm and scanned the entire height of the inks every 30 min 7 times, and the differences of the backscattering and transmission light were detected. The transmittance detector received the light that passed through the dispersion at an angle of 180° with respect to the source, while the backscattering detector received the light scattered backward by the emulsion at an angle of 45°. The emulsion stability was determined with the Turbiscan stability index (TSI) as follows:
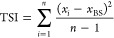
4where *χ*_*i*_ is the average backscattering for each minute during the experiment, *χ*_BS_ is the average *χ*_*i*_, and *n* is the number of scans. TSI is employed for the stability evaluation of emulsions, especially the unstable system. The higher TSI value denotes a less stable system.

#### 2.5.2 Particle Size Measurement

The inks were diluted to a droplet level of about 0.005 wt % with a citrate phosphate buffer (pH 5.6) to avoid the impacts of multiple scattering. Each dispersion was stirred gently at room temperature to ensure the inks were homogeneous. The droplet sizes and particle size distribution of the inks were measured with a laser diffraction device (MS2000, Malvern Instruments Ltd., Worcestershire, UK), measuring the size based on the scattering of a monochromatic beam of laser light (*k* = 632.8 nm). The droplet size was specified as the surface-weighted mean *d*(_3,2_) = (∑*n*_*i*_*d*_*i*_^3^/*∑n_i_d*_*i*_^2^), where *n* is the number of droplets with diameter *d*_*i*_.^4^

#### 2.5.3 Rheological Experiment

The rheological behavior of ink samples was characterized by AR 2000ex rheometer (TA Instruments, New Castle, DE) using a parallel plate geometry (diameter 40 mm, gap 1 mm). The oscillatory strain sweep (0.01–100%, 1 Hz) was performed to attain the limit of the linear viscoelastic region (LVR). The oscillation stress sweep was also conducted from 0.1 to 10 Pa (1 Hz) to the upper limit of stress attainable by the rheometer.^19^ The values of the yield stress of inks were obtained as the crossover point in which elastic modulus equals viscous modulus. Besides, the frequency sweep test (0.1–100 Hz) was accomplished in the viscoelastic region (γ = 0.5%). All measurements were performed at 25 °C. The rheological parameters including storage modulus (*G′*), loss modulus (*G*″), critical strain point, and frequency crossover point (*ω*_c_) were obtained from the relevant software (TRIOS, TA Instruments, West Sussex, UK).^4,20^

To evaluate the steady rheological properties, the inks were presheared with a shear rate of 10 s^–1^ for 5 min at the initial measurement temperature (30 °C) before beginning the experiment cycle. Afterward, the shear stress (τ) was measured as a function of increasing shear rate () from 1–1000 s^–1^. The best constituent rheological equation was selected via statistical analysis, and the rheological variables were measured with the optimum model. Hence, the consistency index, flow behavior index, and yield stress values were obtained by fitting the Herschel-Bulkley model to the data (eq 5).
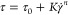
5where τ is shear stress (Pa), τ_0_ is the yield stress (Pa), *K* is the consistency index (Pa s^*n*^), is the shear rate (s^–1^), and *n* is the flow behavior index.

#### 2.5.4 Creep and Creep-Recovery Test

The creep and creep-recovery measurements were performed to evaluate the compliance level in the creep and recovery stages via AR 2000ex rheometer (TA Instruments, New Castle, DE). First, a stress sweep (1 Hz, 0.1–10 Pa) was accomplished (data not shown) to evaluate the oscillatory yield stress (*G′*(*τ*) = *G*″(*τ*)), and then the obtained shear stress values were considered as about 50% of the yield stress. The inks were moved to a parallel-plate geometry with a diameter of 40 mm and a 1 mm gap at 25 °C. The creep measurement included the application of stepwise constant shear stress within the LVR area, from 0 to 500 s, allowing the evaluation of the deformation of the sample between these time intervals. With regard to the recovery phase, the applied stress was rapidly removed (τ_applied_ = 0.0 Pa) and the recovery values were recorded for a further time of 500 s at the same temperature in the creep phase.^4^ The calculated strain and recovery were considered as creep compliance and creep-recovery compliance (*J*) (eq 6). The creep-recovery percentages of inks were then obtained according to eq 7:
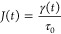
6
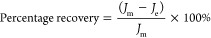
7where *J*(*t*) (Pa^–1^) is creep compliance, γ is the measured strain, *t* is time, τ_0_ is the constant applied shear stress, *J*_m_ (Pa^–1^) is the maximum creep, *J*_e_ (Pa^–1^) is the equilibrium creep compliance after recovery.

#### 2.5.5 Three Interval Thixotropy Test (3ITT)

The 3ITT contained a three-step shear rate test, where the first one comprised a steady shear rate to recognize the ink reference stage without interrupting the microstructure with a fixed shear rate of 1 s^–1^ for 400 s. This was followed by the second interval, in which a steady shear rate of 80 s^–1^ for 200 s was used to terminate the microstructure of the ink. The third interval included a similar assessment condition as the first interval, gaining the reversible restructuration (speed and degree of recovery) of Pickering emulsions.^4^

### 2.6 Printing Process

The 3D printing process of SPI-based inks was conducted through an extrusion-based system (nScrypt-3D-450, nScrypt, Orlando, FL), which is suitable for the fabrication of 3D printed objectives with different shapes and geometries. The system was connected to a syringe pump (PHD Ultra; Harvard Apparatus Holliston, MA), which acted as a pressure system to provide a precise extrusion flow rate of 0.40 mL min^–1^. A specific phrase (BOKU) was modeled using computer-aided design software (AutoCAD; Autodesk Inc., San Rafael, CA) and converted to an STL file. The print paths were provided through the creation of the G-code files to control XYZ direction instruction of the printer, developing by the open-source CAM software Slic3r (slic3r.org, consulted on March 2021) from the STL file. The prepared printable SPI-based inks were carefully put into the stainless-steel cartridge with a volume of 10 mL. The filled cartridge was stirred through a Vortex mixer (Fisher Scientific, Ontario, Canada) for 10 min to eliminate the air bubbles from the ink. To evaluate the impact of the replacement of oil by grafted microbiosurfactant variants on the printing performance, the settings for the printing were adjusted based on different preliminary trials. The layer height was set at 1 mm, proposing that the nozzle tip was elevated by that value upon completion of the fabrication of each layer, continuing until the suitable 3D architectures were printed. The height of the tip was increased by 1.1 mm after the deposition of each layer. The number of deposited layers was 10 and the width of the tip was 1 mm. After printing, the printed constructs were enclosed with a specific aluminum specimen box and stored at 4 °C to prevent dehydration. Table S1 in Supporting Information summarizes the printing settings used to examine the printability of SPI-based inks.

### 2.7 Characterization of 3D Printed Objects

#### 2.7.1 Printing Performance Assessment

The printing quality of the 3D printed objects as affected by the replacement of oil by the grafted microbiosurfactant variants was carried out. The 3D printed structures were moved to a specific chamber (20 × 20 × 20) cm^3^ for taking photos using a digital camera (Alpha 7M3 E-Mount, Full-Frame Mirrorless, 24.2 MP, Sony, Tokyo, Japan). Three replicates of printed constructs were accomplished by determining the line width and layer number through a digital caliper (Mitutoyo, Absolute Digimatic, Tokyo, Japan).^5,20^

#### 2.7.2 Textural Properties

The textural parameters of 3D printed meat analogues were determined from a force-deformation plot using a texture analyzer (TA.XT-plus, Stable Micro Systems, Godalming UK). The printed samples were cut into cylindrical shapes that have a dimension of 30 mm in diameter and 10 mm in thickness and then were compressed with a cylindrical probe (75 mm diameter) based on the texture profile analysis (TPA) method. The texture analyzer was adjusted at 5 mm s^–1^ with a compression distance of 8 cm and a peak force of 15 N. The height of the first force peak on the TPA curve is considered as the hydrogel hardness. The cohesiveness was obtained by the ratio of the positive force region after the second compression phase during the first compression phase. The gumminess was determined from the multiplying hardness in the cohesiveness value. The springiness was considered as the height recovered upon the period between the end of the initial compression and the start of the second compression.^5^ The chewiness was finally measured as gumminess × springiness. All TPA parameters were measured using the Exponent Lite software (ver. 6.1.4, TA.XT-plus, Stable Micro Systems, Godalming UK).

#### 2.7.3 Lubrication Properties

To evaluate the lubricant properties of the 3D printed objects, tribology evolution was performed using a ring-on-plate tribo-rheometry (TA Instrument, New Castle, DE) on a rough hydrophobic surface of 3 M Transpore Surgical Tape 1527-2 (3 M Health Care, St Paul, Min), which was the state to have a comparable surface roughness (*R*_a_ = 31.5 μm) and wettability to the human tongue.^5^ A half-ring rheometry was utilized to provide the refill of material between the two solid surfaces. The tape was cut in a square form and compacted tightly on top of the lower plate rheometry. After each measurement, the tape was replaced and the instrument was cleaned with deionized water. The extent of 3D printed samples was sufficient to cover the surface of the substrate offered a thin film. To mimic the sensory investigation process, the normal forces of 2 N were used to denote the adequate normal force employed upon oral processing.^5^ Moreover, the oral condition was mimicked at a temperature of 37 °C. Each 3D printed object was pre-sheared at a speed of 0.02 s^–1^ for 2 min, and after that equilibrated for 1 min before each measurement. The human tongue was reported to move at a speed of 200 mm s^–1^.^5^ Therefore, the tribology assay in the current study was within this range. Afterward, the increasing rotational speed ramp was set from 0.01 to 200 mm s^–1^ with the attainment of 25 points per decade. The values of coefficient of friction (CoF) were determined as the friction stress (*σ*_F_) proportion to the normal stress (*σ*_N_), described by eq 8:
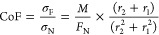
8where *M* is torque (Nm) and *F*_N_ is normal force (N), *r*_1_ is ring inner (14.5 mm), and *r*_2_ is the outer radius (16 mm).

Moreover, the coefficient of friction plotted versus the increasing sliding speed as follows:
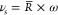
9

Here, *ν*_s_ is the sliding speed (mm s^–1^), *R* is the mean of ring inner and outer radius, and ω is the controlled rotational speed (rad s^–1^).

### 2.8 Statistical Analysis

All instrumental experiments were carried out as triplicate determinations and the mean and standard deviation of the data were reported. Analysis of variance (ANOVA) was utilized for the determination of the main effects of the examined independent factors and their interactions on the instrumental and sensory data. Duncan’s multiple range test was applied to separate means of data when significant differences (*P* < 0.05) were observed.

## 3 Results and Discussion

### 3.1 Characterization of MCC–Polyphenol Conjugates

#### 3.1.1 Grafting Degree

**Scheme 1:**
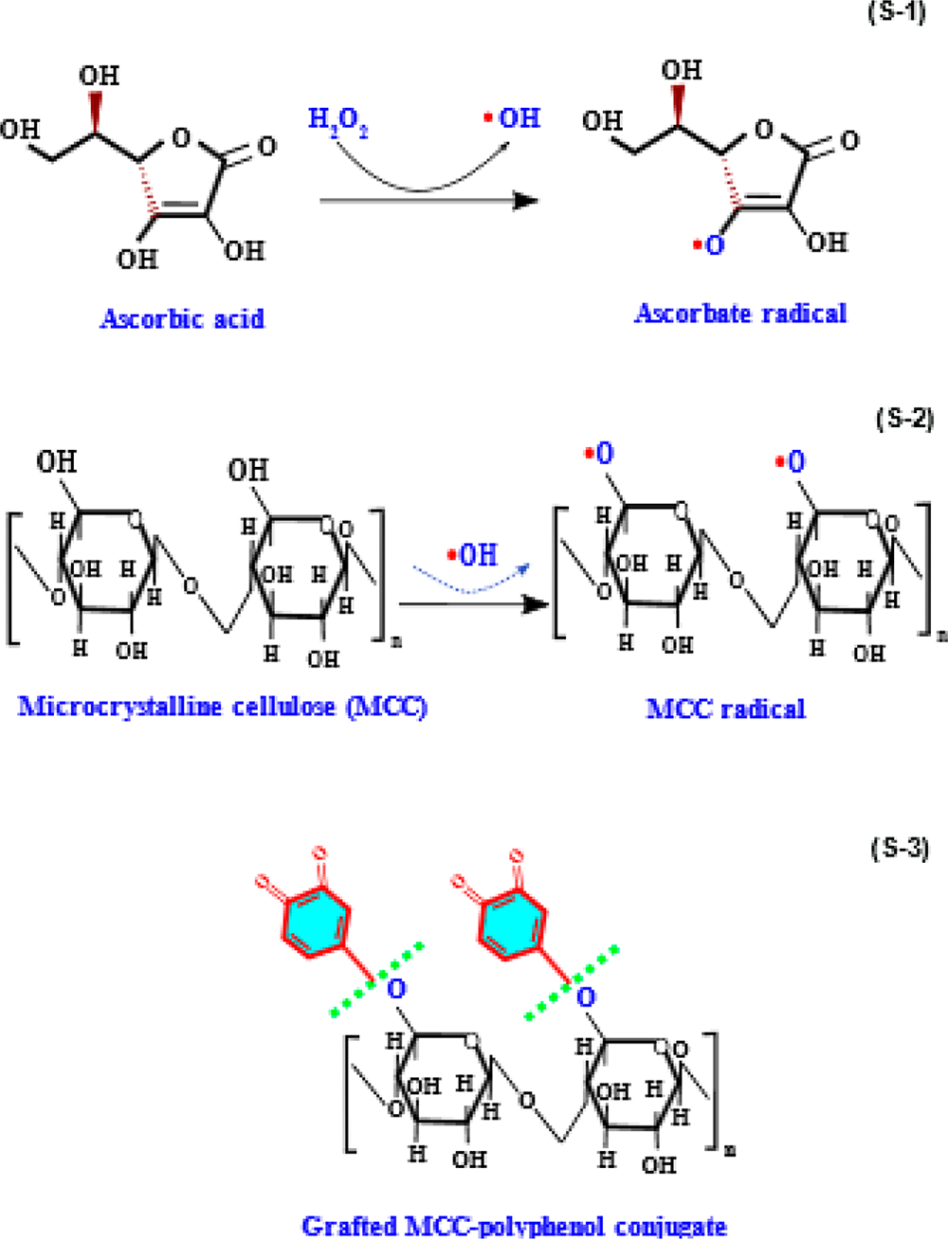
Possible Reactional Pathway of Grafted MCC–Polyphenol Conjugates upon by Free-Radical Grafting Method

The functionality of MCC–polyphenol conjugates, including antioxidant property and surface activity feature, greatly relates to the extent of grafted polyphenol fragments onto the MCC backbone. In the present work, the facile and sustainable free-radical grafting procedure used the hydrogen peroxide/ascorbic acid redox pair as the reaction initiator. As Scheme 1 shows, the reaction between hydrogen peroxide and ascorbic acid induces ascorbate and hydroxyl (**·**OH) radicals (Scheme 1, S-1). Afterward, the hydroxyl radicals induced by the interaction among redox initiator compounds attack the susceptible groups in the MCC backbone, including hydroxyl or carboxyl groups, generating radical species on the MCC (Scheme 1, S-2). Finally, polyphenols in the immediate area of the reactive groups accept the MCC radical, resulting in the development of modified MCC–*g*-polyphenols conjugate (Scheme 1, S-3).

**Figure 1:**
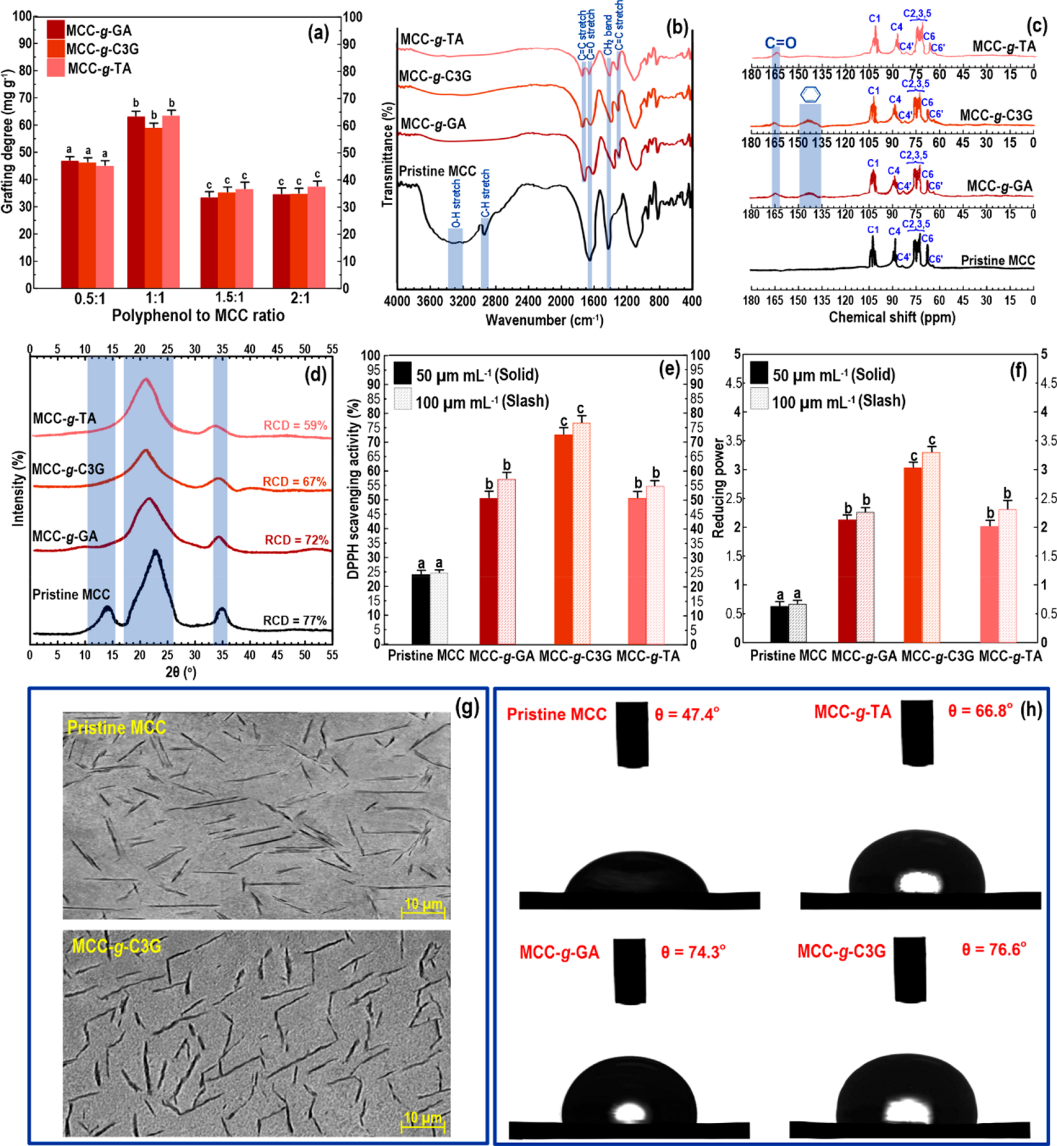
Characterization of grafted MCC–polyphenol conjugates: (a) grafting degree, (b) FTIR, (c) solid-state ^13^C NMR, (d) XRD, (e) antioxidant properties, (f) reducing power, (g) TEM micrograph, and (h) water contact angle. In the case of grafting degree and bioactive features, the means inside each column with various letters (a–c) are significantly different (*P* < 0.05) according to Duncan’s test.

The polyphenol content is a critical parameter for the functionality of grafted MCC–polyphenol conjugates, since it prominently affects the bioactivity properties and emulsifying performance of grafted microconjugates. Generally, the polyphenol content in the resultants is increased by increasing the proportion of polyphenols to MCC.^21^ To develop the optimum MCC–polyphenol conjugates, the grafted microconjugate variants including a different mass proportion of polyphenol variants (GA, TA, and C3G) to MCC (0.5:1, 1:1, 1.5:1, and 2:1) were obtained. Then, the degree of grafting was measured by evaluating the polyphenol amounts. From Figure 1a, with increasing mass ratio up to 1:1 the grafting degree increased significantly (*P* < 0.05). Afterward, a subsequent reduction was found as the mass ratio reached 1.5:1 and beyond to 2:1. It was reported that the quantity of biomacromolecular radicals developed by the constant level of the redox pair initiator (i.e., ascorbic acid/H_2_O_2_) can be comparable in the grafted microconjugates.^22^ The improvement of the grafting degree might be a result of the increase of the polyphenol ratio in the critical ratio. Though, after the critical range, the additional quantity of free polyphenol molecules might prevent the grafting process, resulting in a decrease of grafting degree. Liu et al.^23^ and Hu et al.^22^ reported a similar result, in which the grafting degree was increased up to a certain level of polyphenol to biomacromolecule ratio (0.5:1), and beyond that notably reduced. Thus, the grafted MCC-*g*-GA, MCC-*g*-TA, and MCC-*g*-C3G samples with a mass ratio of polyphenols to MCC of 1:1 were selected as the optimum grafted microconjugates for the following instrumental characterization.

#### 3.1.2 FTIR Assay

The FTIR experiment was accomplished to verify the grafting of the MCC structure after introducing each polyphenol variant. The FT-IR spectra of pristine MCC, MCC-*g*-GA, MCC-*g*-TA, and MCC-*g*-C3G are illustrated to recognize the possible molecular interactions (Figure 1b). A typically pronounced vibration of pristine MCC presented typical bands of cellulose I cantered around 2500–3750 cm^–1^ and 700–1800 cm^–1^. A band at 3380 cm^–1^ could be associated with stretching of −OH groups. Moreover, the representative bands may be comprised as C–H stretch vibration (2953 cm^–1^), carboxyl groups asymmetric stretching vibration (1640 cm^–1^), and methylene symmetrical bending (1435 cm^–1^).^4^

Compared to pristine MCC, the −OH stretching at 3380 cm^–1^ was reduced in all grafted polyphenolic conjugates, representing the grafting reaction was happened at the hydroxyl groups of the MCC structure. Besides, the C–H stretch vibration of −CH_3_ (2953 cm^–1^) disappeared, signifying a possible hydrogen interaction occurred between the hydrogen of −OH or −CH_3_ of the MCC backbone and oxygen of the hydroxyl group of polyphenols.^24^ Additionally, the absorbance peaks of asymmetric stretching vibration of −COOH (1640 cm^–1^) groups and methylene bend (1435 cm^–1^) shifted to the lower wavenumbers. These absorbance peaks became weaker regarding MCC-*g*-C3G, signifying the development of more stable conjugate. Furthermore, there was a new vibration peak at about 1721 cm^–1^ in MCC–polyphenol conjugates because of the C=C stretching. This shows an ester bond was developed through the interaction of hydroxyl groups of MCC and hydroxyl group of polyphenols.^22^ Finally, MCC–polyphenol conjugates showed the emergence of a strong absorbance band at about 1417 cm^–1^. This peak may be associated with the C=C stretching in the aromatic ring, confirming the polyphenols grafting onto the MCC backbone structure.^22^

#### 3.1.3 Solid-State ^13^C NMR

Figure 1c shows the typical ^13^C NMR spectra of pristine MCC and MCC–polyphenol conjugates. Concerning the pristine MCC, the NMR spectrum of cellulose type I, one of the most crystalline types of cellulose known, was detected together with those of amorphous cellulose material.^4^ The peaks from left to right are assigned to the carbons in the d-anhydroglucose units of cellulose. The pristine MCC showed some characteristic NMR bands including C1 (105.1 ppm), C4 (89.1 ppm), C4′ (82.7 ppm), C6 (64.5 ppm), and C6′ (63.8 ppm) with a crystallinity index of about 86%. After the development of grafted MCC–polyphenol conjugates, no noticeable change was detected in the crystalline areas of MCC. However, there was an emergence of a new peak between 135 and 150 ppm resulting from the aromatic ring of phenolic acids.^25^ This is in agreement with the absorbance band at 1410 cm^–1^ obtained from the FTIR experiment. It should be noted that the absence of this characteristic peak in MCC-*g*-TA proposed that the NMR signal might be saturated with a high amount of bulk cellulose, signifying TA coating on MCC was minimal.^25^ Besides, a relatively apparent band appeared in the NMR spectrum of all MCC–polyphenol conjugates at about 165 ppm, associating with the resonance of the C=O peak. This band is in accordance with the C=O stretching at 1640 cm^–1^ obtained from the FTIR assay. Additionally, the measured crystallinity indices revealed that there was a slight change in crystallinity of pristine MCC (about 84%) upon the grafting treatment (∼85%).

#### 3.1.4 XRD Pattern

To further identify the conjugation of MCC with polyphenol variants, the crystallographic structures of grafted MCC–polyphenol conjugates were determined by XRD. The diffractogram of pristine MCC showed the presence of cellulose type I with noticeable peaks at 2θ = 14.2° (*d*_001_ = 5.5 Å), 2θ = 23.0° (*d*_001_ = 4.9 Å), and 2θ = 35.5° (*d*_001_ = 4.1 Å) (Figure 1d). In this case, the RCD (obtained by eq 1) of pristine MCC was detected to be 77%, which was somewhat lower than its crystallinity degree measured by NMR spectroscopy.^4^ The diffractogram of MCC–polyphenol conjugates resulted in the disappearance of the characteristic peak locating around 2θ = 14.2°. This suggests the development of intermolecular interaction between polyphenols and MCC in the interhelical structure. In this regard, the RCD of MCC-*g*-TA and MCC-*g*-GA was dropped to the levels of 72% and 67%, respectively. This denotes a decline in the intensity of crystalline reflection of MCC and also a decrease in the intensity of its main diffraction peak. As Figure 1d exposed, the intensity of diffraction peaks of MCC more decreased regarding MCC-*g*-C3G with a notable reduction in the RCD to 58%. In this context, the pronounced MCC peak (2θ = 23.0°) converted into the diffused peak, representing the loss of crystallinity. On the other hand, the pronounced peak of 2θ = 23.0° in the MCC slightly shifted to about 2θ = 20.5°. This shows an increase in the layer spacing from *d*_001_ = 4.9 Å (2θ = 23.0°) to *d*_001_ = 5.3 Å (2θ = 20.5°). As stated before, there was an interaction between functional groups of polyphenols and MCC, which resulted in the changes of molecular structure and/or unfolding MCC as a result of the grafting process.

#### 3.1.5 Antioxidant Activity and Reducing Power

Different in vitro chemical-based investigations according to evaluations of radical scavenging effect toward ferric reducing capacity and DPPH radicals were made to measure the antioxidant capacity (see also Supporting Information, Section S.1). Figure 1e shows the pristine MCC provided the minimum DPPH scavenging effect than all the grafted MCC–polyphenol conjugates. The scavenging activity of MCC–polyphenol conjugate variants on the DPPH free radicals was noticeably higher than the pristine MCC. In this regard, the MCC-*g*-TA and MCC-*g*-GA more strongly quenched the DPPH radical in the dose-dependent way, which showed almost similar scavenging activity. GA and TA have emerged as strong antioxidant agents and efficient apoptosis-inducing agents,^20^ then grafting GA and TA onto MCC could rationally develop a versatile antioxidant compound with a promising therapeutic property. In the current work, the MCC-*g*-C3G offered the highest antioxidant activity. The grafting of C3G onto the MCC backbone developed polyphenol oxidation and oligomerization. This enlarges the conjugated system of MCC-*g*-C3G and contributes to a rise in the electron-donating strength. Then, this product with a swelling supramolecular matrix can possibly quench the free radicals more efficiently than the GA and TA.

Another imperative index to evaluate the antioxidant activity of a compound is reducing power.^18^Figure 1f shows the reducing power activity of pristine MCC and different MCC–polyphenol conjugate variants. The pristine MCC was distinguished with a poor reducing power property. In contrast, the reducing power of MCC-*g*-TA was stronger than pristine MCC (*P* < 0.05) and equal to MCC-*g*-GA (*P* > 0.05). This was not surprising, as GA and TA are well recognized for their antioxidant ability through the active hydrogen-donating ability.^18^ The data in Figure 1f also specified that the reducing power was more noticeably increased upon the development of MCC-*g*-C3G, proposing grafting of C3G on the MCC considerably enhanced the antioxidant activity of pristine MCC. In this case, the grafting of C3G produced a high-molecular-weight species that could offer a stable system, effectively grabbing the free radicals than the GA and TA.

#### 3.1.6 TEM Evaluation

The pristine MCC and MCC–polyphenol conjugates were subjected to TEM analysis to monitor the morphological properties of particles. The TEM micrographs exposed that the particles in the pristine MCC were in the micron range, which was found to be in the range of about 2–20 μm (Figure 1g). During free-radical grafting, these microparticles were agglomerated to form larger particles with greater dimensions regarding MCC-*g*-C3G (TEM micrographs of MCC-*g*-TA and MCC-*g*-GA not shown). While the surface of the pristine MCC appeared fairly smooth, the MCC–polyphenol conjugates displayed a more barbed nature (Figure 1g). The larger particles with barbed areas along the MCC conjugates might specify that amorphous regions have undergone different degrees of substitution upon grafting reaction.^26^ This observation is also in agreement with FTIR and ^13^C NMR spectroscopy data, where some new peaks emerged in the amorphous regions of grafted MCC–polyphenol conjugates.

#### 3.1.7 Contact Angle

The wetting feature is the capability of a liquid to maintain contact and spread on the surface of a solid.^19^ In many cases, contact angle investigation can be applied to appraise surface hydrophobicity. Figure 1h shows the contact angle images of pristine MCC and grafted MCC–polyphenol conjugate variants. As Figure 1h illustrated, there was a strong interaction between water and the pristine MCC surface compared to modified MCCs. This suggests that typically pristine MCC shows a more hydrophilic character.^19^ The free-radical grafting greatly enhanced the surface hydrophobicity of MCC. In this case, the water contact angle of MCC film was increased by 19.4° and 26.9° regarding MCC-*g*-TA and MCC-*g*-GA, respectively. The surface of MCC-*g*-C3G was found to have the highest hydrophobicity, with a contact angle of θ = 76.6°. The improvement in the surface hydrophobicity of MCC–polyphenol conjugates effectively may be caused by the development of covalent linkages with MCC. This resulted in the additional consumption of free OH groups of MCC. Therefore, there is a notable decrease in the hydrophilic nature of MCC as confirmed by a higher value of contact angle.

### 3.2 Characterization of SPI-Based Pickering Emulsions

#### 3.2.1 Particle Diameter and Polydispersity Index

**Figure 2:**
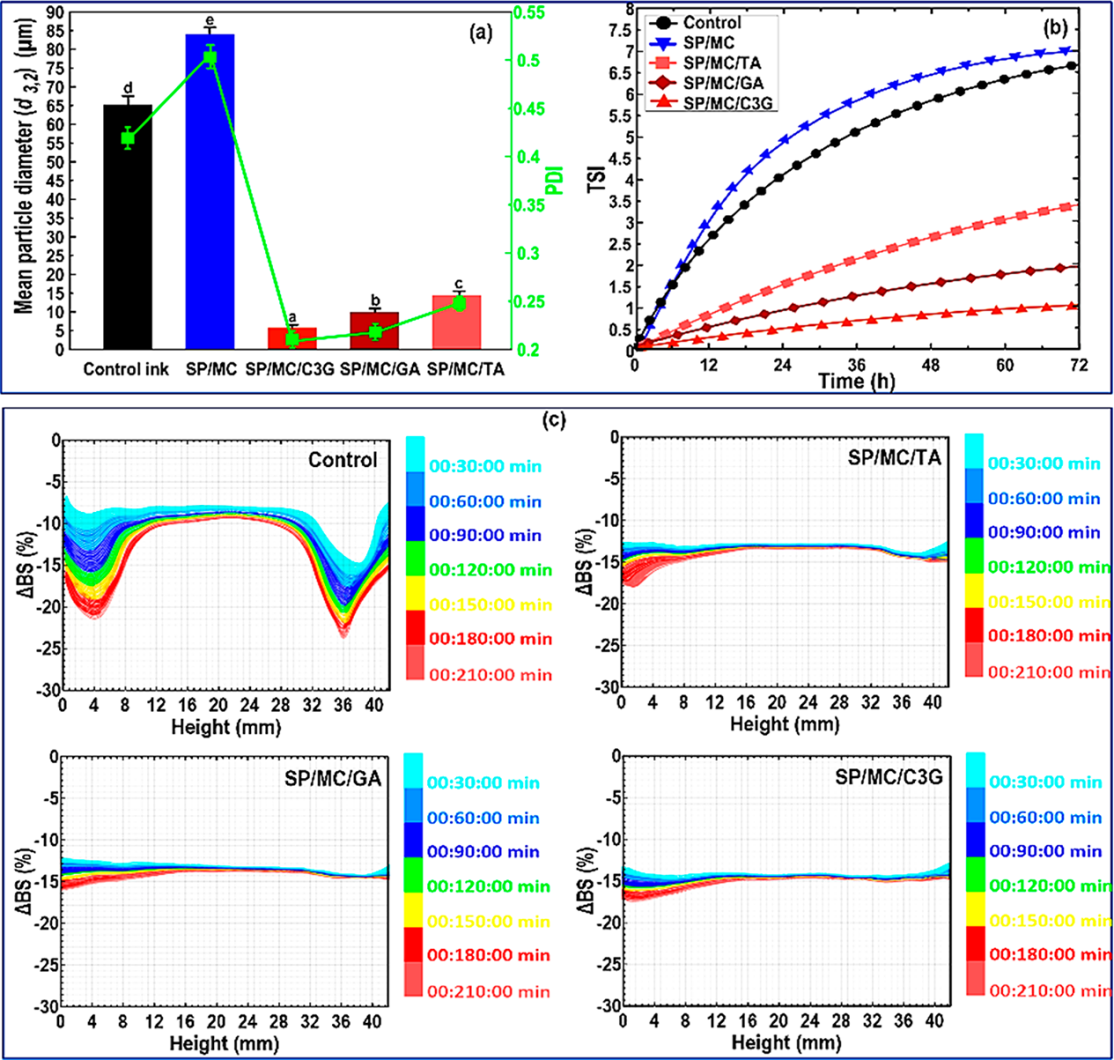
Characterization of SPI-based Pickering emulsions formulated with pristine MCC or different grafted MCC–polyphenol conjugates: (a) (*d*_3,2_) and PDI parameters, (b) TSI parameter, and (c) (ΔBS) profiles. In the cases of (*d*_3,2_) and PDI, the means inside each column with various letters (a–e) are significantly different (*P* < 0.05) according to Duncan’s test.

The effect of oil replacement with pristine MCC or grafted MCC–polyphenol conjugates variants on the (*d*_3,2_) and PDI of SPI-based emulsion (after 48 h storage) is illustrated in Figure 2a. The (*d*_3,2_) and PDI parameters of control SPI-based emulsion were detected to be 65 μm and 0.42, respectively. The oil replacement by pristine MCC in the SPI-based emulsion (*i.e*., SP/MC ink) increased both (*d*_3,2_) and PDI parameters (Figure 2a). It was reported that pristine MCC shows a lack of adsorption at the oil–water interface due to its high hydrophilicity, limiting its application as an effective stabilizer.^4,5^ In contrast, the oil replacement by all MCC–polyphenol conjugates led to the development of highly efficient emulsions with a mean particle diameter less than 15 μm (Figure 2a). In this case, the mean droplet diameter of SP/MC/GA ((*d*_3,2_) = 10 μm) and SP/MC/C3G inks ((*d*_3,2_) = 6 μm) was lower than SP/MC/TA system ((*d*_3,2_) = 14 μm) owing to more hydrophobicity (Figures 1h). The average particle size is proven to be a paramount feature to evaluate the emulsion stability. It was stated that a smaller particle size enhances the emulsion stability against coalescence/flocculation thanks to stronger repulsion that prevents aggregation between droplets.^27^ In the present study, the grafted microconjugates seemed to effectively coat the droplets, preventing the occurrence of coalescence.^4^ On the other hand, the PDI of control ink considerably decreased after the inclusion of grafted microconjugates. Furthermore, the PDI of Pickering emulsion droplets in the emulsion gel containing MCC-*g*-C3G (PDI = 0.21) was somewhat lower than those of including MCC-*g*-GA (PDI = 0.23) and MCC-*g*-TA (PDI = 0.26) (Figure 2a), showing a highly stable emulsion after 48 h with uniform particle size distribution.^27^ Accordingly, the MCC-*g*-C3G, being more hydrophobic (Figure 1b,c,h) and having less charge, offered an efficient stabilizing effect on the emulsion particle size and also improved the uniformity of reduced-fat SPI-based ink.

#### 3.2.2 Stability of Pickering Emulsions

The global TSI parameter, accounting for diverse storage processes of emulsion (particle coalescence and settling processes), is commonly employed to determine emulsion stability. The TSI value of various inks was measured and recorded as a function of time (Figure 2b). In this case, the TSI curve tended to increase upon oil replacement by pristine MCC in the SPI-based emulsion. This is likely due to the high surface charge density and hydrophilic character of unmodified MCC, reducing its tendency for adsorption at O/W interfaces, and therefore poor emulsion stability.^4^ However, the stability of soy-based ink was enhanced after introducing grafted microconjugates into the system. The reduced-fat Pickering emulsion gel stabilized by MCC-*g*-C3G was more stable than those of MCC-*g*-GA and MCC-*g*-TA, correlating well with its higher hydrophobicity (Figure 1h). The better emulsion stability provided by grafted microconjugates may also be associated with increasing the viscosity of reduced-fat emulsions, and also increasing the internal friction of the fluid so that the TSI was reduced and the stability of the system became better.^27^

The stability analysis of different ink variants after 210 min of storage at 25 °C was also performed based on the transmission (*ΔT*) and delta-backscattering (ΔBS) profiles (Figure 2c). The ΔBS of control and SP/MC (not shown) inks overall decreased with time. This signifies there was a progressive increase in the particle size resulted from flocculation/coalescence, which is also consistent with (*d*_3,2_) results and TSI data. In addition, there was an increase in the width of peaks in the bottom and the top of the tested bottle with time (Figure 2c). This could be associated with the fact that the large droplets migrated owing to the difference in oil and water densities (creaming phenomenon). Regarding reduced-fat Pickering emulsion gels containing grafted microconjugates, the particle size was quite a bit smaller than the control. Consequently, the emulsion creaming process was considerably delayed due to the network structures, forming by the Pickering emulsion droplets covered with more hydrophobic particles. Moreover, the ΔBS profile of the inks at the bottom slowly reduced for Pickering emulsions stabilized by grafted microconjugates. Therefore, the incorporation of grafted microconjugates retarded the phase separation in the reduced-fat inks. Compared to MCC-*g*-TA, the MCC-*g*-GA and MCC-*g*-C3G could more efficiently enhance the stability of SPI-based ink against coalescence (Figure 2c). It was expected that the creaming of oil droplets slowed in the systems with the smaller particle size.^27^

#### 3.2.3 Flow Curve of SPI-Based Pickering Emulsions

**Figure 3:**
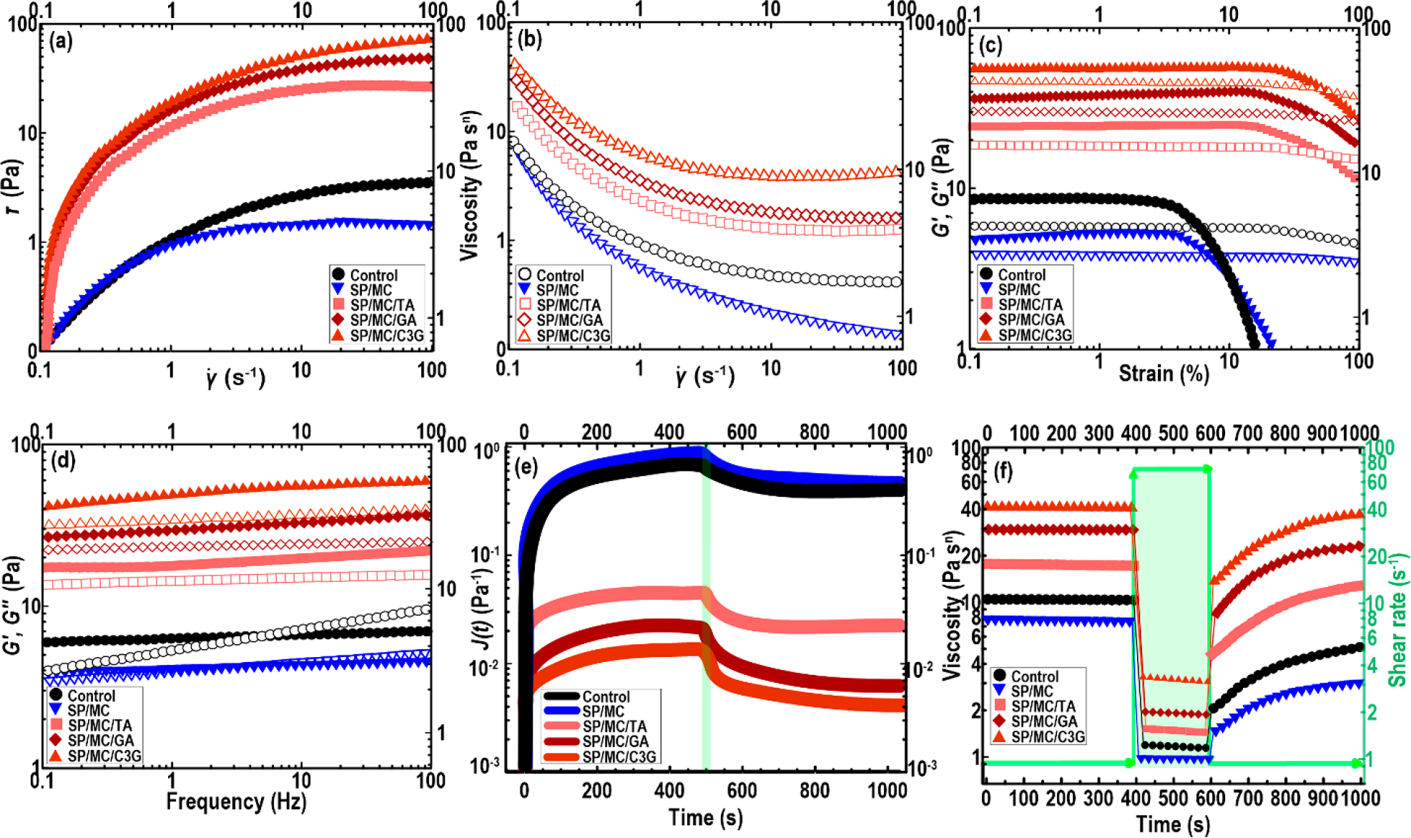
Changes in the shear stress (a) and viscosity (b) as a function of shear rate. Strain (c) and frequency (d) sweeps curves of Pickering emulsion gels. In this case, the solid symbols denote *G′* and open symbols refer to *G″*. (e) Creep and creep-recovery plots. (f) 3-ITT curve of different Pickering emulsion gels.

In extrusion 3D printing, the study of flow properties provides crucial information regarding printability and printing performance. It was reported that the printable inks with well-defined shear-thinning and strong viscoelastic features can be effectively printed in a variety of complex geometric structures.^2^Figure 3a shows the changes in the shear stress of ink samples as a function of shear rate, which shows that our ink formulations flowed with a characteristic non-Newtonian behavior. The flow behavior index of SP/MC ink, derived by fitting the Herschel-Bulkley model, was higher than the control ink (Supporting Information, Table S2), showing poor shear-thinning character. This result could be associated with the lack of pristine MCC existing at the oil–water interface due to its higher hydrophilic nature and larger electrostatic charge. In contrast, extensive shear-thinning behavior was observed regarding reduced-fat inks containing grafted microconjugates (Supporting Information, Table S2), which is representative of highly concentrated or flocculated systems.^27^ This type of *pseudoplasticity* is consistent with the flow behavior of colloidal dispersions comprising aggregated particles. As the shear rate is increased, the droplet flocs or biopolymer aggregates become increasingly deformed and disrupted, thus decreasing the resistance to flow and increasing shear-thinning. In the current work, the extent of shear-thinning was highly dependent on the type of MCC–polyphenol conjugates, being much more pronounced for the inks containing MCC-*g*-GA and MCC-*g*-C3G (Supporting Information, Table S2).

From Figure 3b, the viscosity of all ink samples reduced appreciably as the shear rate increased. In this regard, SP/MC/GA, SP/MC/TA, and SP/MC/C3G inks showed more viscous behavior than control and SP/MC samples. The grafted microconjugates with an amphiphilic nature linked the SPI-covered droplets together, thus developing flocs that surrounded some continuous phases inside them. Accordingly, there was an increase in the effective volume of disperse phase, increasing the viscosity.^27^

The initial yield stress of SPI-based emulsion was decreased after the oil replacement by pristine MCC (Supporting Information, Table S2), as did the apparent shear viscosity (Figure 3a). This could be related to the lack of network formation (essential to offer yield stress) due to the higher hydrophilic nature and a larger electrostatic charge of pristine MCC. Moreover, there might be a possible agglomeration between SPI and pristine MCC, which decreased the effective volume of both biopolymers at the oil–water interface for surface coverage. In contrast, rheological data revealed that the yield stress increased in the Pickering emulsion gels formulating by grafted MCC–polyphenol conjugates. These effects may be associated with the fact that the grafted microconjugates tend to form aggregated networks due to their higher hydrophobicity, in that way increasing the elasticity of the system.^27^ Furthermore, the observed increase in yield stress might have been a consequence of the increased association of the biopolymeric molecules (e.g., surface-modified MCC and SPI) or ionic particles due to electrostatic screening or ion-bridging effects.^4^

#### 3.2.4 Strain Sweep

Viscoelasticity is an important design parameter for Pickering emulsions used in 3D printing.^2^ The inks with strong viscoelastic properties can be efficiently extruded to yield a continuous 3D structure with high shape-fidelity. The viscoelastic properties of the SPI-based emulsion gels were characterized through the strain sweep investigation and recorded as elastic (*G′*) or viscous (*G″*) moduli versus strain in Figure 3c. The oscillation test revealed that all the inks showed a largely gel-like character, where the *G′*(*γ*) was greater than *G″*(*γ*) during a range of strain amplitudes evaluated. As also observed in Figure 3c, the SP/MC ink showed noticeably lower *G′*(γ) than control SPI-based ink. The presence of pristine MCC with higher hydrophilic nature and larger electrostatic charge inhibits the network formation (essential to get *G′*).^4,27^ Compared to control ink, the viscoelastic moduli became larger in the reduced-fat Pickering emulsion gels including grafted microconjugates, where the elastic modulus dominated viscous one values for a wide range of strains (<40%). This reveals the elastic or gel-like behavior of these emulsions. This further highlighted the importance of increasing the effective size of the aggregated oil droplet clusters and the presence of aggregated networks on the rheological properties of the emulsions.^16^ In comparison, the ink formulated with MCC-*g*-C3G showed the highest *G*′(γ) among all reduced-fat Pickering emulsion gels. This results from the formation of a structured and mechanically strong emulsion due to the higher hydrophobicity of MCC-*g*-C3G (Figure 1h).

The length of LVR can be considered as the structural strength of a system as the strong networks could stay in LVR over greater strain amplitude compared to the weaker structure.^4,16^ Consistent with oscillatory strain sweep results, control and SP/MC inks showed a more limited LVR, showing a critical strain magnitude of 3.6% and 2.7%, respectively. On the other hand, the Pickering emulsion gel prepared by MCC-*g*-C3G offered the highest critical strain value (59.6%) (Figure 3c). Though beyond the LVR, the elastic and viscous moduli should be evaluated with caution, nonetheless *G′*(*γ*) values gradually decreased beyond the critical amplitude, regardless of ink type. This demonstrates the inks from a quasi-gel structure shifted to a quasi-viscous phase (Figure 3c). After this transition, there was a reduction in the *G′*(*γ*) or *G″*(*γ*), showing that the stiffness of emulsions was reduced as the strain amplitude increased. The main reasons for the decreased viscoelastic properties could be related to (*i*) deformation and disruption of droplet flocs into the smaller clusters and (*ii*) the disentanglement of the biopolymeric structures, e.g., surface-modified MCC and SPI.^4^

#### 3.2.5 Frequency Sweep

Additional pieces of information about the impact of pristine MCC or grafted microconjugates on the dynamic viscoelastic features of the Pickering emulsion gels were obtained using oscillatory frequency sweep measurement (Figure 3d). The values of *G′*(*ω*) were appreciably higher than *G″*(*ω*) regarding control and SP/MC inks, signifying that the SPI-based emulsion was predominantly elastic. In these cases, a crossover (*ω*_*c*_) was noted between *G′*(*ω*) and *G″*(*ω*) curves (i.e., *G*′ = *G″*) at the higher frequencies (>3 Hz). This crossover indicates that the gel-like character of the system was changed to liquid-like behavior.^28^ Regarding reduced-fat inks containing grafted microconjugates, the trend of *G′*(*ω*) or *G″*(*ω*) was only weakly dependent on the frequency, and there was no crossover in the range of frequencies tested. These points specify that SP/MC/GA, SP/MC/TA, and SP/MC/C3G inks developed a stable network structure, which could be ascribed to a change in their structure or the state of droplets aggregation. Compared to control and SP/MC inks, the *G′*(*ω*) or *G″*(*ω*) values of Pickering emulsion gels were higher, which is consistent with the formation of gel-like emulsion held together by noncovalent interactions.^4^ Among the reduced-fat inks containing grafted microconjugates, the values of *G′*(*ω*) or *G″*(*ω*) were the highest in the ink prepared with MCC-*g*-C3G. This again may have been due to the ability of MCC-*g*-C3G to develop a strong grafting affinity with SPI, leading to a more hydrophobic conjugate. Particles of such conjugated MCC (when adsorbed onto the droplet surface) result in the formation of stronger bonds between the MCC-covered droplets.

#### 3.2.6 Creep-Recovery Test

The polymeric-based inks with a thixotropic character can effectively maintain the spatial shapes upon 3D printing, particularly offering beneficial effects in the 3D printing of intricate architectures.^2^ The thixotropic property of inks was carried out through assessment of Pickering emulsion gels viscoelasticity via the creep and creep-recovery measurements (Figure 3e). In the creep-recovery assessment, constant stress is employed, where the system is quickly deformed. This imposes a strain on the system, continuing to increase at a reducing rate as a function of time. Once the constant stress is released, the strain declines and may approach back to zero-value, depending on the sample properties.^4,16^ As can be observed in Figure 3e, the creep compliance of the reduced-fat Pickering emulsion gels made from grafted microconjugates was lower than those of the control and SP/MC inks. In general, the creep compliance peaks of SP/MC/GA (*J*(*t*) = 0.23 Pa^–1^) or SP/MC/C3G (*J*(*t*) = 0.13 Pa^–1^) inks were 41- and 73-fold lower compared to control and SP/MC inks (*J*(*t*) ≈ 9.5 Pa^–1^), respectively. Compared to SP/MC/GA and SP/MC/C3G inks, the SP/MC/TA ink (*J*(*t*) = 0.52 Pa^–1^) showed a moderately weaker structure due to a larger creep compliance peak (Figure 3e). The enhancement of the elastic properties could be owing to the formation of a stronger aggregated system because of the higher hydrophobic nature of modified MCCs, and hence stronger bonds between their particles. Alternatively, the inductions of phenolic dimers and trimers or polyphenol oxidation/oligomerization offer longer chain length with greater molecular weight, requiring additional stress for the Pickering emulsion gels to be deformed.

Regarding the recovery phase of the creep test, a higher relative recovery can be associated with greater elasticity, offering a gel-like character.^16^ The relative recovery capacity was reinforced in the reduced-fat Pickering emulsion gels including grafted microconjugates. Compared to control and SP/MC inks (∼46%), a premiere recovery percentage was detected concerning SP/MC/TA (70%), SP/MC/GA (85%), and SP/MC/C3G inks (89%). This signifies an enhanced elasticity with the formation of a more structured system. The creep-recovery test proposed that the development of a highly recoverable structure in Pickering emulsion gels containing grafted microconjugates, where their original network matrices were quickly restored after the breakdown.

#### 3.2.7 3ITT Experiment

As mentioned before, thixotropic properties, as important parameters in 3D printing applications, can assume the geometrical retention, therefore inhibiting the geometry instability and avoiding discontinuities.^2^ The 3ITT can be considered an effective method to carry out a quick shear rate to mimic the impacts of extrusion force upon 3D printing.^4^ The viscosity of printable ink variants was measured and plotted as a function of shear rate and time (Figure 3f). Regarding the first shearing interval, the Pickering emulsion gels prepared by grafted MCC–polyphenol conjugate variants showed considerably higher viscosity than control or SP/MC inks. In the current study, the control or SP/MC inks presented lower viscosity recovery with a value of about 42% and 38%, respectively. This might be because of irreversible microstructural failure, in which these emulsions exhibited a very soft network owing to the improper elasticity. In contrast, viscosity recovery of SP/MC/GA, SP/MC/TA, and SP/MC/C3G inks was measured to be about 70%, 72%, and 88%, respectively. The higher thixotropy regarding these reduced-fat Pickering emulsion gels might be associated with the reinforcement of SPI-based emulsion structure upon by oil replacement by grafted microconjugates. This offers inks resistance to the prompt deformation. These results are consistent with the steady and dynamic rheological investigations, in which the structured Pickering emulsion gels including grafted microconjugates showed a robust solid-like behavior.

### 3.3 Characterization of 3D Printed Constructs

#### 3.3.1 Printing Performance

**Figure 4:**
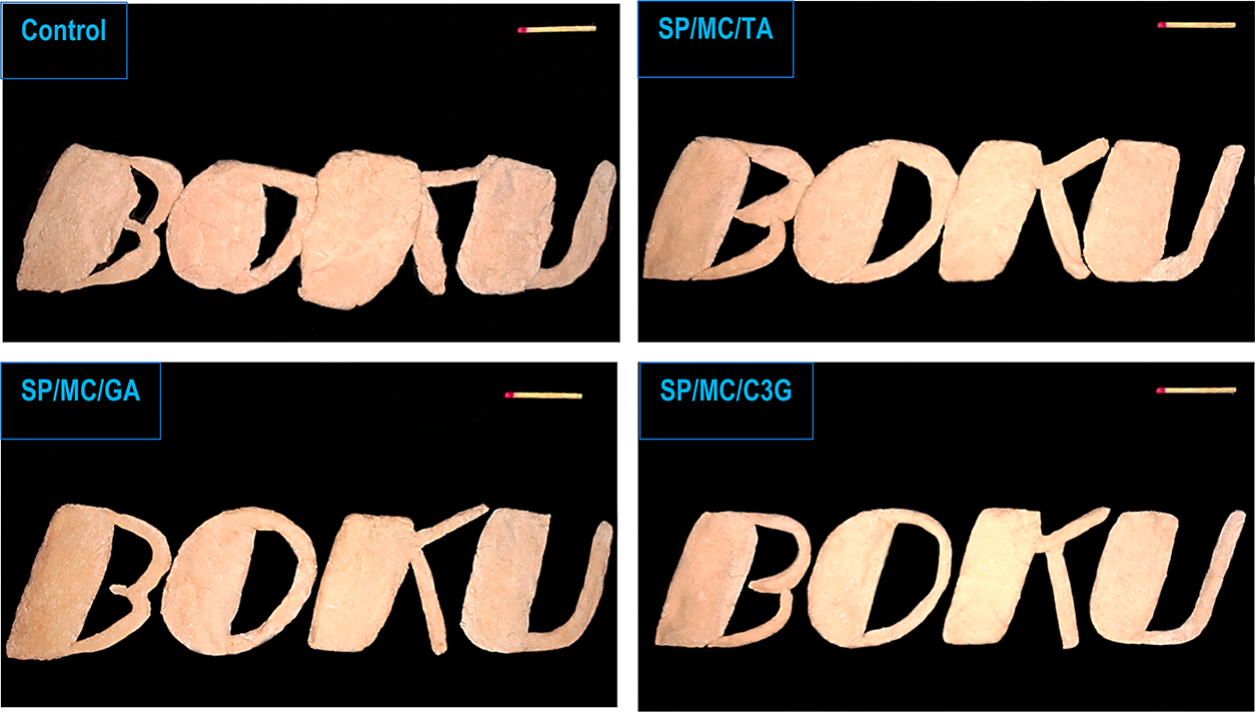
Photographs of 3D printed architectures produced by different kinds of Pickering emulsion gels. The scale bar is 4 cm.

The effect of the replacement of oil by different MCC–polyphenol conjugate variants on the printing quality of SPI-based objects is shown in Figure 4. The printing process of all ink samples was continuous, and the layers could adhere to each other. The control (with no MCC–polyphenol conjugate) and SP/MC (not shown) inks had the less reversible network to be efficiently extruded from the nozzle, where the obtained 3D printed object was dry with an uneven structure. This makes these samples more likely to be susceptible to collapse and cracking (Supporting Information, Section S.3). Overall, the control and SP/MC (not shown) inks could not take an appropriate shape upon the extrusion-based printing process. This causes the 3D printed control and SP/MC constructs to not reach the desired structural strength supporting the subsequently deposited layers, therefore leading to poor shape-fidelity and compressed deformation. As can be seen in Figure 4, the printing performance of SPI-based emulsion enhanced when oil was replaced by the MCC–polyphenol conjugate variants, in which the 3D-printed constructs showed a high resolution with precisely defined geometries (see also Supporting Information, Section S.4). In this case, the SP/MC/TA ink was effectively squeezed out, and the 3D printed layers could effectively adhere to the previous layer during extrusion and deposition. Concerning SP/MC/GA and SP/MC/C3G, these reduced-fat inks could also be effectively extruded, where they preserved their originally designed shape during deposition. Among the reduced-fat Pickering emulsion gels, the SP/MC/C3G emulsion was the most appropriate ink for the 3D printing process, because it was printed with a well-defined geometry shape. In this regard, this ink showed a robust solid-like structure with the desired thixotropic feature, giving 3D printed SP/MC/C3G an improved shape retention ability and good adhesion to previously deposited layers.

The mechanical strength and printing precision can be determined by layer number and line width of 3D printed objects, respectively.^2^ As the 3D printed control and SP/MC construct presented a poor resolution with a weak 3D structure, it is reasonable to have a thick line width with small layer numbers (Supporting Information, Table S3). In contrast, the inks formulated with grafted microconjugates offered the printed constructs with higher layer numbers and thinner line width. The formation of a solid-like network in Pickering emulsion gels containing grafted microconjugates could justify their shape fidelity and precise geometry. In these cases, the higher elastic modulus with greater thixotropic behavior could result in the improved spatial resolution and shape fidelity of 3D printed objects.^5,20^

#### 3.3.2 Textural Features of Printed Constructs

The texture is a physical feature relating to the rheological and structural characteristics of materials, which can offer useful information regarding the qualitative properties and shelf life of 3D printed products.^5,20^ In this sense, the TPA assay can then support a better understanding of the effect of the grafted microconjugate on the developing 3D printed structures. From Table S3 (Supporting Information), the textural parameters of printed SP/MC were lower than those of the printed control. This can be related to a weaker mechanical strength of SP/MC ink. The textural data showed that the hardness (as an indicator of system rigidity) of printed control objects was increased after the addition of MCC-*g*-TA (i.e., printed SP/MC/TA), whose extent from 12.6 N significantly increased to 15.3 N (*P* < 0.05) (Supporting Information, Table S3). Regarding 3D printed SP/MC/GA and SP/MC/C3G, the hardness parameter was increased by 70% and 120%, respectively. The intermolecular junctions between biopolymeric chains could be strengthened in the presence of grafted microconjugates, limiting their elongation and arrangement.^29^ This could likely lead to more alignment of polymeric chains during the development of 3D structures, inducing a more compact and stronger 3D printed network. The TPA test revealed that cohesiveness parameters could not be affected by the addition of grafted microconjugates (*P* > 0.05). Regarding springiness (referred to as elasticity) and gumminess, the grafted microconjugates significantly increased these parameters (*P* < 0.05). According to TPA measurement, the chewiness value notably increased by about 110%, 320%, and 400% regarding 3D printed SP/MC/TA, SP/MC/GA, and SP/MC/C3G, respectively (Supporting Information, Table S3). The higher values of chewiness could be associated with the interaction of hydrophilic domains of grafted MCC and SPI through hydrogen bonding. This develops the intramolecular bonds among the polymeric backbones, subsequently strengthening the 3D structure. For the purposes of the current work, the 3D printed objects with firmer, more chewable, and strong gel-like structures could be beneficial to consider a desired edible 3D structure.

#### 3.3.3 Tribological Properties of 3D Printed Constructs

**Figure 5:**
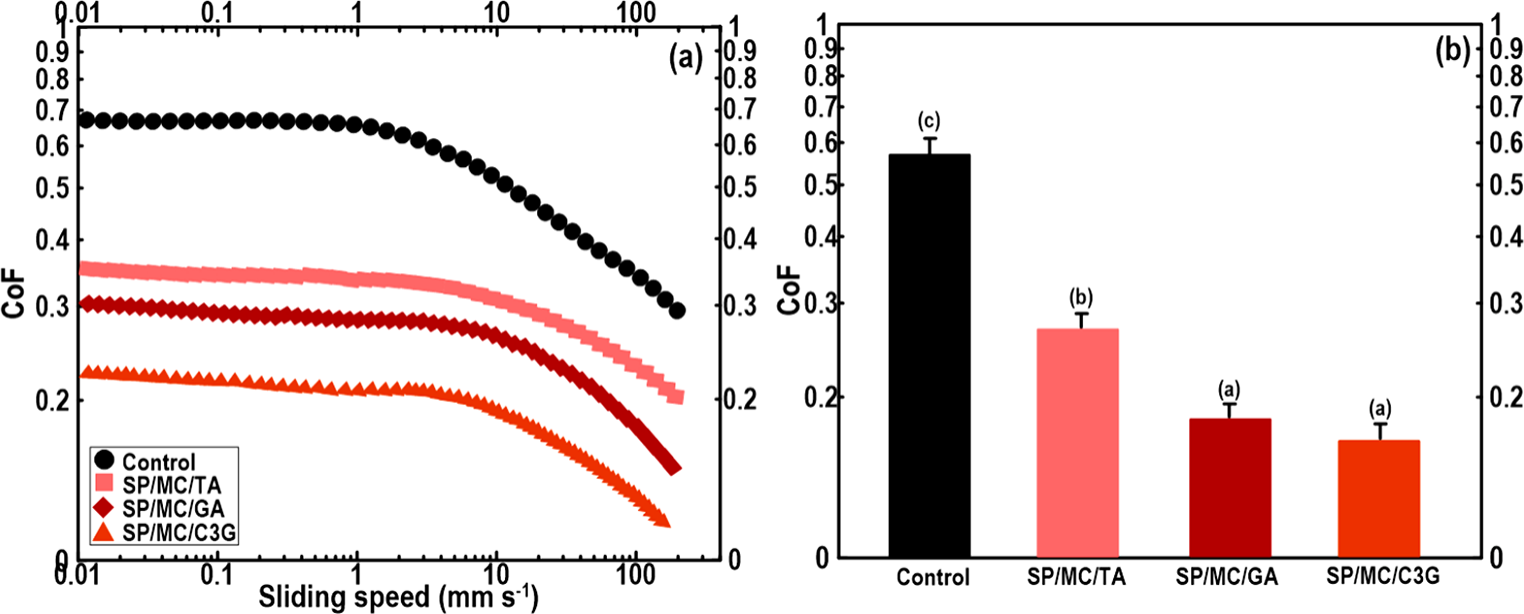
(a) Friction plots of different 3D printed meat analogues. (b) Measured friction of coefficient. The means that each column with different letters (a–c) is significantly different (*P* < 0.05), Duncan’s test.

The lubricating properties of 3D printed objects were investigated by measuring the friction coefficient (CoF) obtained by ramping up the entrainment speeds. Figure 5a shows the Stribeck curves (ramping up) obtained for different 3D printed objects. At the low speed (<0.2 mm s^–1^), the corresponding plots of 3D printed samples did not change with sliding speed, representing a boundary regime. As the sliding speed was increased (0.2 > mm s^–1^), a decrease in the friction was noted, which the “boundary” region shifted to the “mixed” area. This designates a friction behavior conversion from “surface–surface contact” to “lubricating fluid starts to separate the surface”.^30^ The phenomena likely relate to a purely elastic response below the critical friction force of the 3D printed meat analogue, thus increasing the CoF. Obviously, a greater elasticity together with the wetting ability indicates that the systems were outside the boundary area where the surface features are dominant.^5^

The difference in the plot tendency is easily recognized when comparing the 3D printed control and SP/MC (not shown) objects with reduced-fat 3D printed constructs containing grafted microconjugates. A sharper peak in the ramping up curve can be related to a system with larger particles. Compared to the 3D printed control and SP/MC objects, the mean friction of printed SP/MC/TA, SP/MC/GA, and SP/MC/C3G was decreased, being roughly half as small in magnitude (Figure 5b). This suggests that the grafted microconjugates are able to promote particle entrainment, indicating more lubrication properties than the 3D printed control and SP/MC. This lower CoF could be a sign of the considerably greater elastic modulus of the reduced-fat inks containing grafted microconjugates. This result is in accordance with the steady and dynamic rheological experiments. A stronger gel-like structure is difficult to deform as compressed with vertical forces among two contact surfaces. This would increase the separation of bumpy surfaces and decreasing CoF. High lubrication of the 3D printed objects formulated with Pickering emulsion gels caused slippage during extrusion 3D printing. Thus, increasing CoF by the addition of grafted microconjugates contributed to the reduced slippage during the 3D printing process. Among reduced-fat 3D printed objects, the 3D printed SP/MC/GA and SP/MC/C3G showed the lowest CoF. These samples with greater viscoelasticity avoid close contact among the surfaces through the development of the thin-film, resulting in a lower CoF.^20,31^

## 4 Conclusion

The engineering of an antioxidative low-fat printed meat analogue needs the use of an effective multifunctional Pickering emulsion gel with enhanced bioactivity and emulsion stability. The challenge was to covalently graft different types of polyphenols, including gallic acid, tannic acid, or cyanidin-3-*O*-glucoside onto microcrystalline cellulose side chains. Hence, three grafted microconjugates, bearing polyphenol variants bonded to the microcrystalline cellulose backbone, were successfully synthesized. The instrumental data confirmed the effective grafting of different polyphenol variants onto the microcrystalline cellulose backbone, resulting in the development of an antioxidative grafted microconjugate. Rheological behavior analyses suggested that the reduced-fat soy-based emulsion gels containing grafted microconjugates showed a *pseudoplastic* behavior, endowing higher viscosity, strong gel-like structure, and a thixotropic feature with antioxidant properties. In this scenario, cyanidin-3-*O*-glucoside provided an efficient soy-based emulsion gel, decreasing droplet size and improving the emulsion stability against coalescence. After the printing process, the reduced-fat 3D printed constructs formulated with the grafted microconjugate variants presented an enhanced printing performance with excellent structural stability. The printed constructs also offered a reinforced mechanical strength with a more chewable matrix. Moreover, the lubricity test showed that grafted microconjugates offered a decrease in the friction coefficients. The results described in this work could advantage the fabrication of innovative healthy 3D-printed products containing bioactive ingredients. Future investigations focused on more complex systems will offer many possibilities for custom-made and personalized nutrition through 3D printing and will provide for more fabrication and recognize more inventions.

## Supporting Information Available

Antioxidant activity and reducing power of Pickering emulsion gels (section S.1). Effect of different levels of microconjugate variants on functionally of SPI-based emulsion (section S.2). Particle diameter and polydispersity index (section S.2.1). TSI measurement (section S.2.2). Emulsion stability by vertical laser profiling (section S.2.3). Side view of 3D printed objects (section S.3). The .Stl file of 3D printed sample (section S.4). Printing settings (Table S1). Steady rheological parameters (Table S2). Printing performance results and textural data (Table S3) (PDF)
