# Supplementary material for: Development of an Antioxidative Pickering Emulsion Gel through Polyphenol-Inspired Free-Radical Grafting of Microcrystalline Cellulose for 3D Food Printing
Source: Biomacromolecules. 2021 Oct 1;22(11):4592–605. doi: 10.1021/acs.biomac.1c00896 (PMC8579399; doi:10.1021/acs.biomac.1c00896)
Supplement: Supplementary file 1 — bm1c00896_si_001.pdf [file bm1c00896_si_001.pdf]

Supporting Information for:

Development of an antioxidative Pickering emulsion gel through polyphenol-inspired free-radical grafting of microcrystalline cellulose for 3D food printing

Mahdiyar Shahbazi[†*], *Henry Jäger*[†*], *Rammile Ettelaie*[‡]

†Institute of Food Technology, University of Natural Resources and Life Sciences (BOKU), Muthgasse 18, 1190 Vienna, Austria

‡Food Colloids and Bioprocessing Group, School of Food Science and Nutrition, University of Leeds, Leeds, LS2 9JT, UK

S.1. Antioxidant activity and reducing power of "Pickering emulsion gels"

In addition to the grafted MCC compound, the antimicrobial activity and reducing power of all obtained Pickering emulsion gels (including control SPI, SP/MC, SP/MC/GA, SP/MC/TA, and SP/MC/C3G inks) were performed. Compared to the pristine MCC or grafted MCC-polyphenol conjugates, the antimicrobial activity and reducing power of the respected inks were slightly decreased (Figure S.1). The data exposed that the control SPI-based ink and SP/MC had the lowest DPPH scavenging effect or reducing power, while SP/MC/C3G ink offered the highest antioxidant activities. These results were not surprising, as the incorporation of polyphenols onto MCC could rationally induce a versatile antioxidant property with a promising therapeutic application. These results are also in accordance with those obtained for the grafted MCC-polyphenol conjugates.

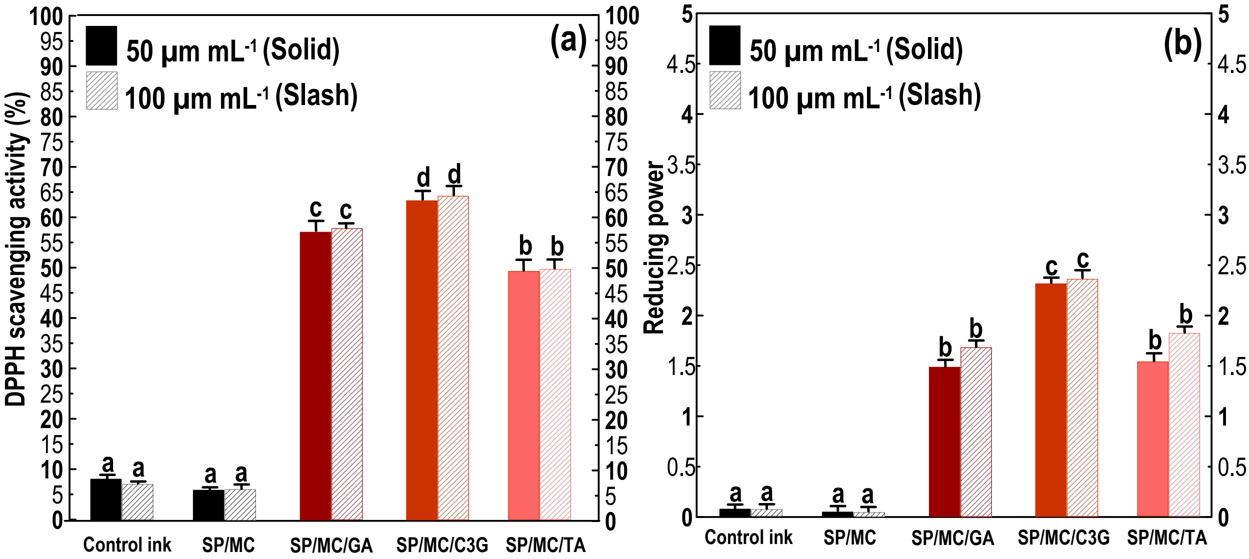

Figure S.1. (a): Antioxidant properties and (b): Reducing power of different emulsion gels. The means inside each column with various letters (a–c) are significantly different ($P < 0.05$) according to Duncan's test.

S.2. Effect of different levels of micro-conjugate variants on functionally of SPI-based emulsion

In the preliminary assays in order to produce 'reduced-fat' Pickering emulsion gels, five different levels from each MCC-polyphenol conjugate variant were incorporated into the control SPI-based emulsion (10 wt.% canola oil, 25.0 wt.% SPI, pH 5.6) to be replaced with the part of its oil, according to the Method stated in the main Manuscript (Section 2.4). In this regard, the levels of 1.05, 2.1, 3.15, 4.2, and 5.25 wt.% of each MCC-polyphenol conjugate (i.e., MCC-g-GA, MCC-g-TA, and MCC-g-C3G) were added to the SPI-based emulsion (10 wt.% canola oil, 25.0 wt.% SPI, pH 5.6) to be replaced with 15, 30, 45, 60, and 75% of the oil in the system, respectively.

Based on emulsion stability data and rheological results, the MCC-polyphenol conjugate variants in the ranges of 1.05-3.15 wt.% did not change the initial mean particle diameter ($d_{3,2}$), poly-dispersity index (PDI), TSI, delta-backscattering, and apparent viscosity parameters. In contrast, the concentration of 4.2 wt.% of MCC-polyphenol conjugates (i.e., 60% oil-reduced ink) significantly affected all these

parameters ($P < 0.05$), showing a high stable emulsion. However, beyond this level (i.e., 5.25 wt.%), the droplet size and TSI value notably increased, promoting some levels of emulsion instability.

In this case, since the results of 60% reduced-fat SPI-based Pickering was affected by the presence of MCC-polyphenol conjugate variants (in the level of 4.2 wt.%), the instrumental results of this sample were discussed.

The detailed physical results for this point have been listed as follows:

S.2.1. Particle diameter and polydispersity index

The initial mean particle diameter (($d_{3,2}$) = 61 µm) and polydispersity index (*PDI* = 0.39) of the droplets-coated SPI (control ink) showed the presence of some flocculated oil droplets having a non-uniform particle size distribution. After the addition of MCC-polyphenol conjugates in the levels of 1.05-3.15 wt.%, the ($d_{3,2}$) and *PDI* of SPI based-emulsion remained unchanged ($P > 0.05$), attributing to the presence of oil droplets' flocculation (Figure S.2). Contrary, the incorporation of more levels of MCC-polyphenol conjugates (4.2 wt.%) lead to the conclusion that the development of extremely effective emulsion as both ($d_{3,2}$) and *PDI* reduced (Figure S.2). However, the ($d_{3,2}$) and *PDI* of SPI based-emulsion considerably increased after the incorporation of MCC-polyphenol conjugates in the content of 5.25 wt.% ($P < 0.05$). This effect may have been caused by the adsorbed layer thickness of SPI and MCC-polyphenol conjugates was possibly inadequate to coverage oil droplets due to agglomeration. This offers a lack of stability via steric repulsions, as particles could not adsorb on the surface of droplets (Shahbazi, Jäger, & Ettelaie, 2021).

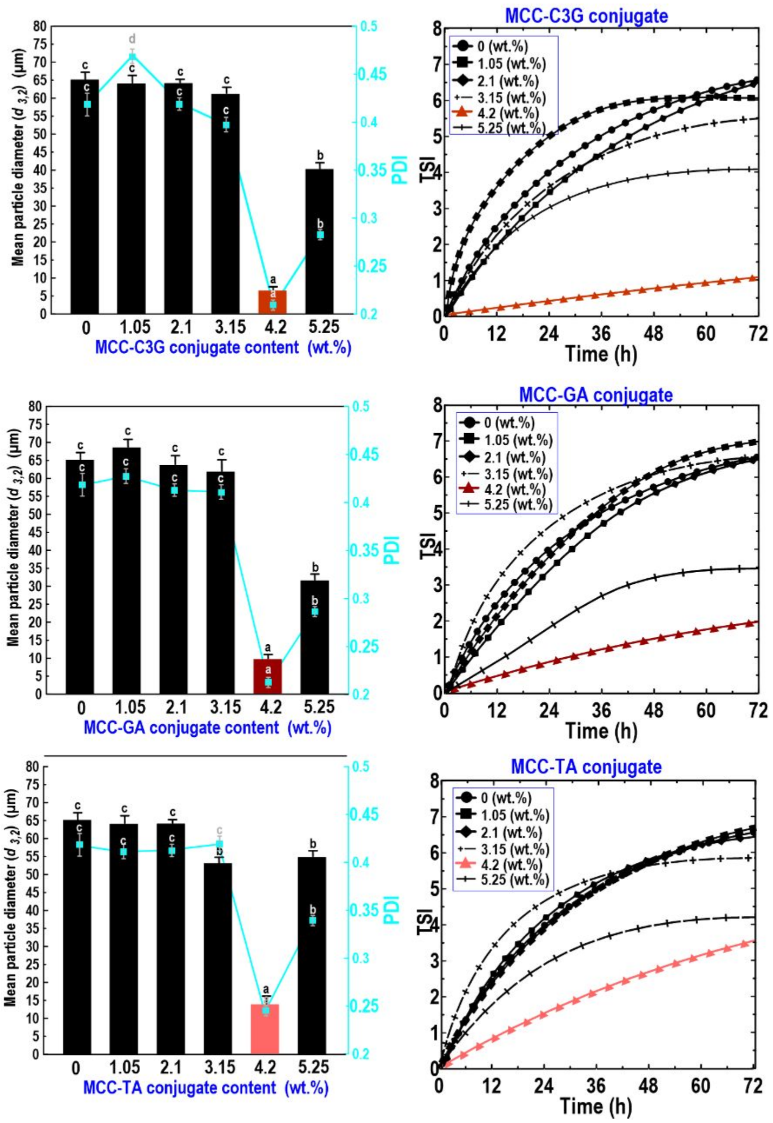

Figure S.2. The ($d_{3,2}$) and PDI parameters of SPI-based Pickering emulsions formulated with different ratios of grafted MCC–polyphenol conjugates. In the cases of ($d_{3,2}$) and *PDI*, the means inside each column with various letters (a–e) are significantly different ($P < 0.05$) according to Duncan's test.

S.2.2 TSI measurement

The *TSI* values of various emulsions were calculated and plotted as a function of time (Figure S.2). Again, the MCC-polyphenol conjugates in the ranges of 1.05-3.15 wt.% did not change the *TSI* parameter of SPI-based emulsion. The MCC–polyphenol conjugate variants in a concentration of 4.2 wt.%, by contrast, developed a stable SPI-based emulsion with a high level of stability. The better emulsion stability provided by grafted micro-conjugates (i.e., MCC-*g*-GA, MCC-*g*-TA, and MCC-*g*-C3G) may be attributed to an increase in the viscosity of the aqueous phase and also increasing the internal friction of the fluid (Shahbazi et al., 2021). Again, the grafted MCC–polyphenol conjugates in the level of 5.25 wt.% produced an important increase in the *TSI* value. This might be attributed to oil droplets' flocculation as a result of introducing a high level of MCC–polyphenol conjugates, where they could not be well-dispersed in the system. Alternatively, the interaction of SPI with MCC–polyphenol conjugates

might decrease the thickness and charge of the lipid droplets. This weakens the flocculation stability of droplets by decreasing the steric and electrostatic repulsion between them.

S.2.3 Emulsion stability by vertical laser profiling

The stability analysis of soy-based emulsion after the addition of different levels (1.05-5.25 wt.%) of each MCC-polyphenol conjugate after 4 h storage at 25 °C was also performed using vertical laser profiling based on the transmission (*ΔT*) and delta-backscattering (*ΔBS*) profiles (Figure S.3). The *ΔBS* of control emulsion decreased overall with time, signifying an incremental increase in the particle size caused by flocculation or coalescence, which is also according to *TSI* results. Furthermore, the peak widths in the bottom and the top of the tested bottle increased with time. The presence of MCC-polyphenol conjugate variants in the ranges of 1.05-3.15 wt.% did not produce a stable emulsion (Figure S.3). By contrast, the backscattering of the emulsion prepared with 4.2 wt.% MCC-polyphenol conjugates showed a little change in the lower-middle-upper part (20–35 mm), a slight decrease at the top (40–42 mm), and a slight increase at the bottom (0–5 mm) of the measuring bottle (Figure S.3). This result proposes that the long-term physical stability of emulsion containing 4.2 wt.% MCC-polyphenol conjugates, which could be interpreted from the short-term vertical laser profiling assessment. However, the level of 5.25 wt.% MCC-polyphenol conjugates led to a reduction of the *ΔBS* of the bottom and the top layer of SPI-based emulsion over time, signifying important phase separation. This could be associated with the migration of large droplets from the bottom to the top of the bottle owing to the difference of density between oil and water. Moreover, it might emanate from the decreasing the effective volume of the added higher levels of MCC-polyphenol conjugates on account of a possible agglomeration between SPI and modified MCCs. The polar head groups of MCC would be possible to have more interaction with hydrophilic sites of SPI molecules. This may decrease the interaction between SPI molecules and oil, which may make larger droplet sizes.

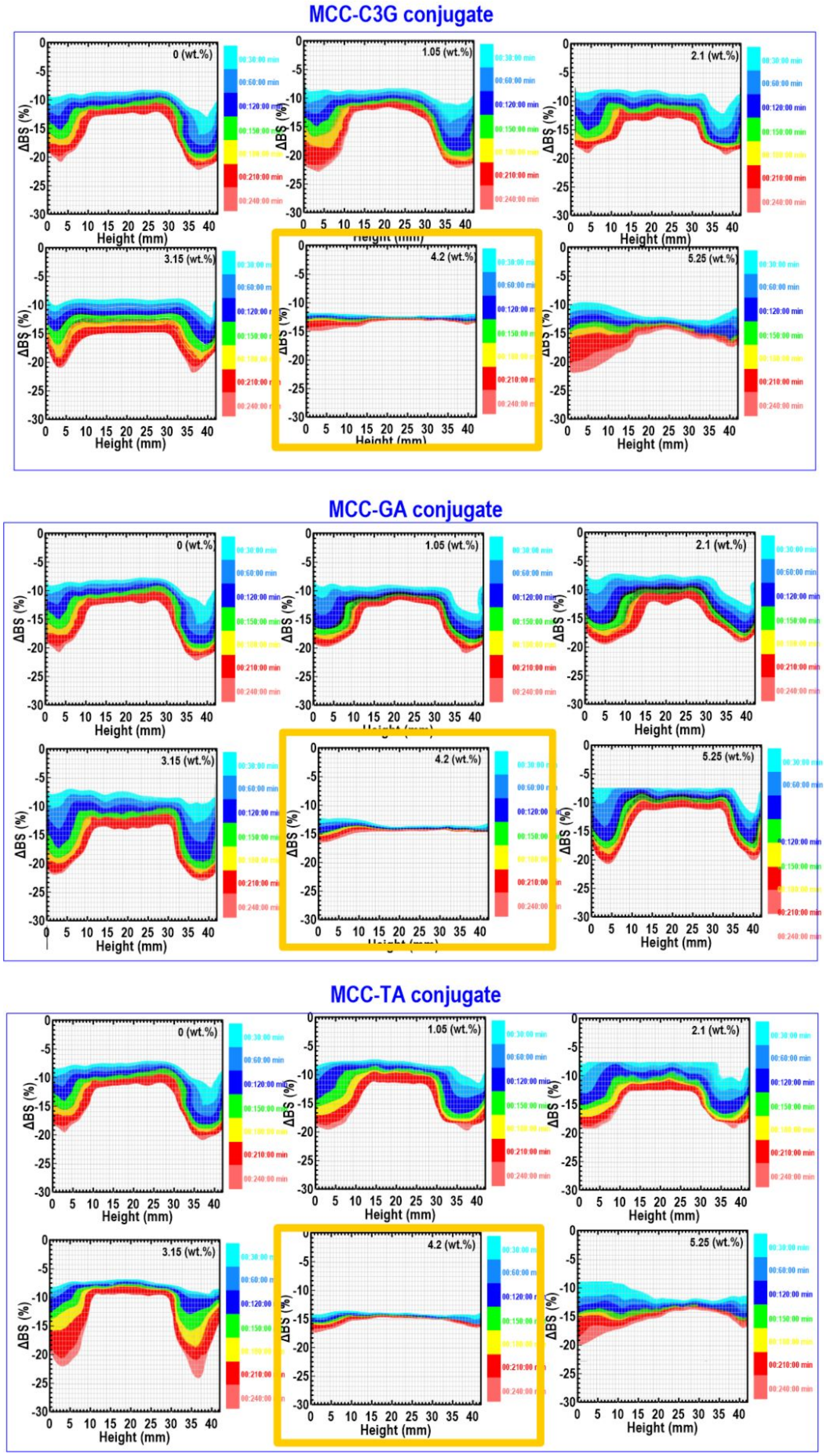

Figure S.3. The (ΔBS) profiles of SPI-based Pickering emulsions formulated with different ratios of grafted MCC–polyphenol conjugates.

S.3. Side view of 3D printed objects

After 4 h of the printing process, the 3D printed structures were moved to a specific chamber (20 × 20 × 20) cm^3 to take the photos from their side view by a digital camera (Alpha 7M3 E-Mount, Full-Frame Mirrorless, 24.2 MP, Sony, Tokyo, Japan) (Figure S.4).

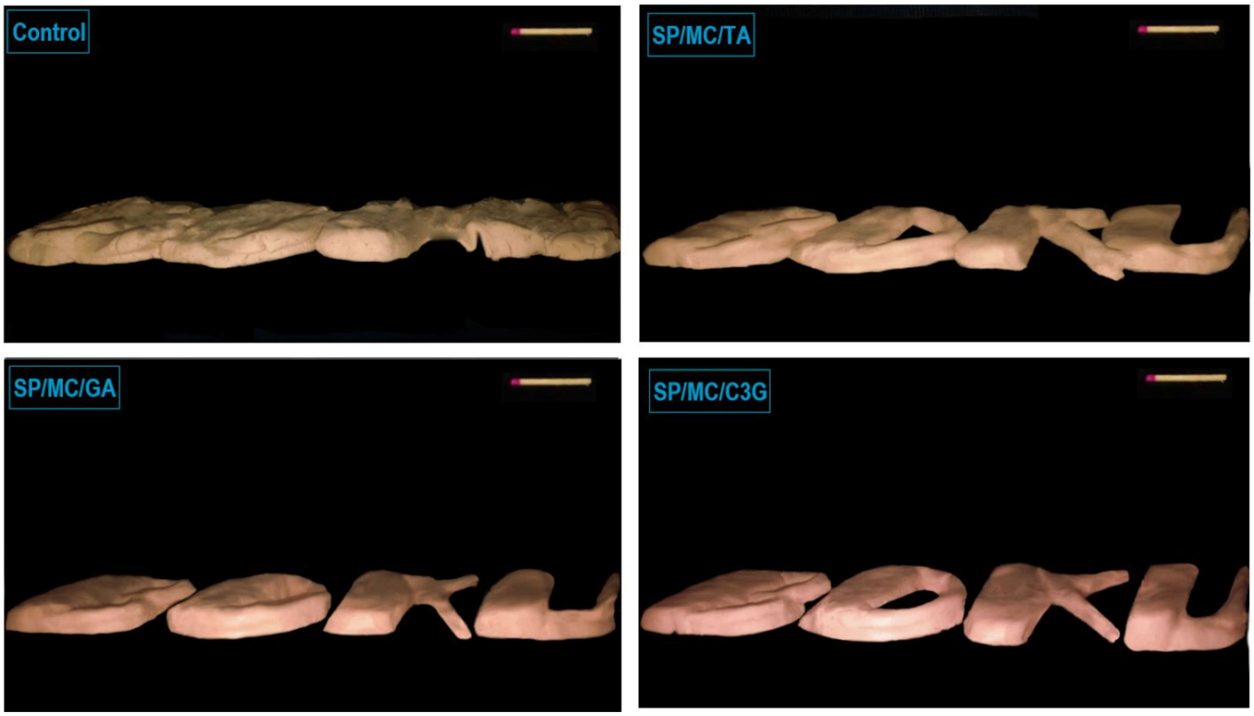

Figure S.4. The printing performance images of different 3D printed objects (side view). The scale bar is 4 cm.

S.4. The .Stl file of 3D printed sample

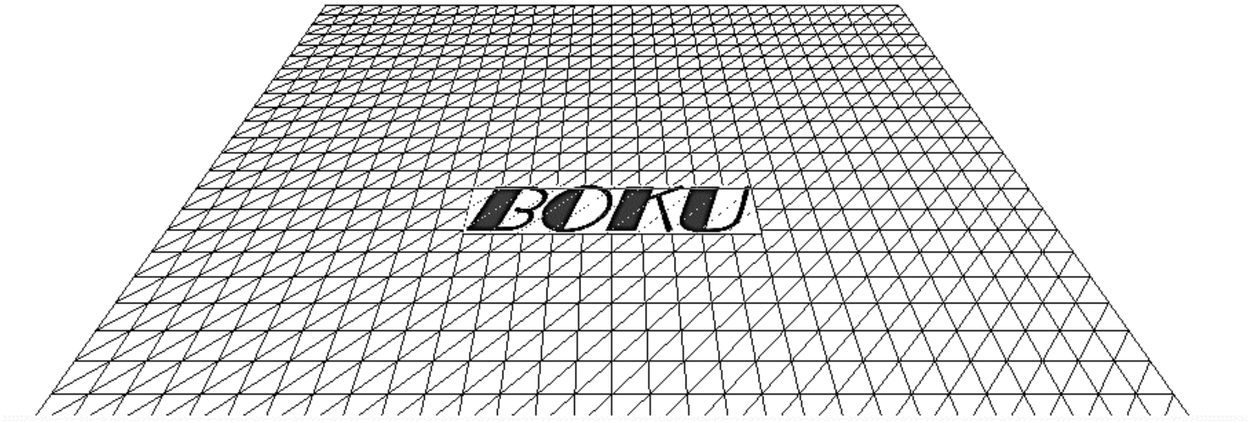

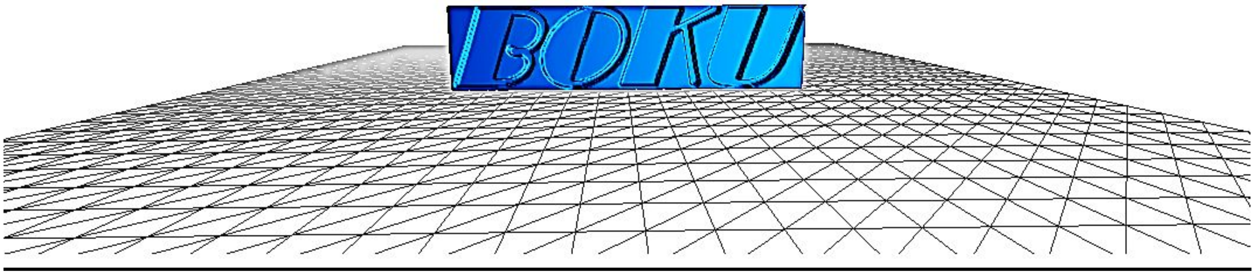

Figure S.5. The .Stl files (dimension of the designed samples was 24.5 cm length × 8.5 cm width × 6 cm height)

Table S1. The printing settings are expressed as Slic3r terms (http://slic3r.com).

Printing adjusting	Sign	Value	Units	Definition
**Nozzle diameter**	D	1.0	mm	Nozzle diameter
**Layer height**	Z	1.0	mm	Layer height
**Extrusion flow speed**	Q	0.40	mL min^{-1}	Continuous extrusion flow rate provided by the syringe pump
**Flow rate**	S	90	%	The volume of ink that passes through the extruder
**Infill velocity**	V	15	mm s^{-1}	Spindle speed during extrusion
**Travel velocity**	V_{travel}	180	mm s^{-1}	The spindle speed of a jump between the end of one extrusion and the next
**Perimeter**	P	10	-	Number of outline layers
**Infill density**	ρ_{infill}	90	%	Quantity of material filling the object

Table S2. The obtained viscosity, flow behavior index, and yield stress of SPI-based ink variants.

Sample	Flow behavior index	Consistency index (Pa s^n)	Yield stress (Pa)	R^2
**Control**	0.873±0.022^d	7.44±0.52^b	1.65±0.22^b	0.982
**SP/MC**	0.997±0.054^e	4.37±0.32^a	0.89±0.13^a	0.982
**SP/MC/TA**	0.616±0.034^c	10.82±0.43^c	5.56±0.75^c	0.973
**SP/MC/GA**	0.556±0.019^b	22.73±0.68^d	6.21±0.18^d	0.994
**SP/MC/C3G**	0.513±0.021^a	27.05±0.88^e	7.37±0.53^e	0.993

$^{a–e}$ Means (three replicates) within each column with different letters are significantly different (P < 0.05), Duncan's test.

Table S3. Summary of printing performance results and textural parameters of 3D printed variants.

Sample	Printing performance		TPA parameters				
	Line width (mm)	Layer number	Hardness (N)	Cohesiveness	Gumminess (N)	Springiness (mm)	Chewiness (N mm)
**Control**	7.1 ± 0.6^{c}	3 ± 1^{a}	12.6 ± 0.6^{b}	0.42 ± 0.01^{b}	5.3 ± 0.2^{b}	4.1 ± 0.3^{a}	21.7 ± 1.9^{b}
**SP/MC**	7.6 ± 0.4^{d}	2 ± 1^{a}	11.2 ± 0.7^{a}	0.39 ± 0.01^{a}	4.4 ± 0.2^{a}	4.3 ± 0.2^{a}	18.8 ± 1.6^{a}
**SP/MC/TA**	4.2 ± 0.4^{b}	6 ± 1^{b}	15.3± 0.3^{c}	0.43 ± 0.03^{b}	6.6 ± 0.3^{c}	4.9 ± 0.3^{b}	32.3 ± 1.3^{c}
**SP/MC/GA**	4.4 ± 0.3^{b}	6 ± 1^{b}	21.4 ± 0.7^{d}	0.42 ± 0.02^{b}	9.0 ± 0.2^{d}	5.1 ± 0.3^{b}	45.9 ± 2.2^{d}
**SP/MC/C3G**	3.8 ± 0.3^{a}	8 ± 1^{c}	27.7 ± 0.9^{e}	0.42 ± 0.04^{b}	11.6 ± 0.4^{e}	5.6 ± 0.2^{c}	64.0 ± 2.1^{e}

$^{a–e}$ Means inside each column with different letters are significantly different ($P < 0.05$), Duncan's test.
